# Fault tolerant and quality of service aware routing algorithm based on priority technique for scalable network on chip architectures

**DOI:** 10.1038/s41598-025-20381-3

**Published:** 2025-10-21

**Authors:** Xiaomo Yu, Ling Tang, Jie Mi, Jiajia Liu, Long Long

**Affiliations:** 1 Department of Logistics Management and Engineering, Nanning Normal University, Nanning, 530001 Guangxi China; 2 College of the Arts, Guangxi Minzu University, Nanning, 530001 Guangxi China; 3 Guangxi Colleges and Universities Key laboratory of Intelligent Logistics Technology, Nanning, 530001 Guangxi China; 4 College of Computer Science and Information Engineering, Nanning Normal University, Nanning, 530001 Guangxi China

**Keywords:** Network on chip, Fault-tolerant routing, Quality of service, TOPSIS, Multi-criteria decision-making, Adaptive routing, Scalability, Latency, Throughput, Hardware overhead, Engineering, Mathematics and computing

## Abstract

Network on Chip (NoC) architectures are essential subsystems for on-chip communication. They use routers and simplified protocols modeled after public data networks to transport packets using complex routing algorithms from their source to their destination. Reliable communication can be severely hampered by component failures, such as malfunctioning routers or cables, which can interrupt packet transfer. Performance may be harmed by the narrow criteria used by traditional fault-tolerant routing algorithms to find reliable routes. In order to improve routing reliability and Quality of Service (QoS) in scalable NoC architectures, this paper suggests a novel, adaptive fault-tolerant routing algorithm that incorporates the Technique for Order of Preference by Similarity to Ideal Solution (TOPSIS), a multi-criteria decision-making technique. The suggested approach dynamically assesses and ranks alternate routes to choose the best ones, even when there are failures, by utilizing path length and density information from nearby nodes. On 8 × 8 meshes with 10% link failures, the approach reduces average delay by ~ 8–12% compared to EDAR and increases throughput by ~ 2–5% compared to EDAR; on application-driven traces, it reduces delay by ~ 5–15% at nearly equal throughput. It reduces energy per flit by around 15–20% compared to EDAR, improves throughput by about 3–4%, and lowers delay by about 8–10% under transient, thermal, and voltage disturbances. The two-stage decision core maintains the improvements on 16 × 16 meshes and reroutes locally in about 3–5 cycles without adding a critical-path cost. Additionally, the approach ensures scalability for large-scale NoC implementations by introducing low hardware overhead. The suggested algorithm is a viable answer for next-generation NoC designs, meeting the requirements of high-performance, dependable, and scalable on-chip communication systems thanks to its combination of fault tolerance, QoS awareness, and resource efficiency.

## Introduction

 Chip networks facilitate communication among several intellectual cores or processing components^1^. The primary objectives of the NoC design approach are to enhance efficiency^2^, minimize latency^3^, and decrease power consumption^4^. The most prevalent communication architecture on a semiconductor is the shared bus system, which offers advantages such as simplicity and minimal implementation overhead^5^. Nonetheless, when the length of the shared bus line expands and the number of components integrated on the chip rises^6^, the parasitic signals along this shared pathway will escalate significantly^7^. Consequently, the augmentation of propagation delay resulting from this phenomenon will restrict the number of processing units that can be integrated into this communication system, thereby diminishing scalability^8^. The limited scalability, significant area overhead for point-to-point communications, and substantial communication delay associated with a shared bus are critical shortcomings of this communication method, prompting designers to adopt the NoC communication approach to mitigate overhead and enhance efficiency^9,10^. The implementation of a novel communication architecture known as NoC facilitates inter-unit communication via packet routing through integrated routers and switches^11^. Scalability, reduced cable length, and an increased number of nodes at the chip level will decrease power consumption and enhance network bandwidth^12–14^.

One of the critical challenges in NoC design is ensuring fault tolerance, as failures in routers and links can significantly impact data transmission^15^. Traditional fault-tolerant algorithms often rely on predefined criteria to identify alternative paths, limiting their adaptability in dynamic network conditions^16^. In contrast, adaptive routing strategies offer greater flexibility by dynamically selecting paths based on real-time network conditions^17^. However, balancing reliability, performance, and hardware overhead remains a significant concern in designing effective routing algorithms.

This paper proposes a QoS-aware fault-tolerant routing algorithm that utilizes the TOPSIS for multi-criteria decision-making. The proposed method ranks potential routing paths by considering factors such as path length and link density, ensuring the selection of an optimal route in the presence of faults. By incorporating a stress value parameter that represents link density within the router, this approach enables efficient congestion management and improves network performance. The proposed algorithm enhances network reliability while maintaining minimal hardware overhead, making it suitable for large-scale NoC implementations.

The rapid advancement of semiconductor technologies has led to an exponential increase in the number of cores integrated on a single chip. This evolution has necessitated the development of high-performance and scalable communication architectures^18^, as traditional shared bus structures suffer from limitations such as excessive power consumption, high propagation delays, and scalability issues^19^. NoCs address these challenges by enabling efficient packet-based communication; however, their reliability remains a critical concern, particularly in large-scale implementations where component failures can significantly disrupt network performance.

Fault-tolerant routing mechanisms play a crucial role in ensuring reliable data transmission within NoCs. A robust fault-tolerant routing algorithm must be able to detect faults dynamically and reroute packets through alternative paths without causing congestion or significant performance degradation^20,21^. This study introduces an innovative routing approach that enhances NoC resilience by utilizing the TOPSIS decision-making method to identify the most suitable routing paths based on multiple criteria. The design of fault-tolerant NoC routing algorithms presents several challenges, including dynamic fault detection, load balancing, scalability, latency, throughput, and deadlock prevention. Identifying faulty components in real-time without excessive hardware overhead is a complex task, and ensuring an even distribution of traffic across the network is essential for preventing congestion and performance degradation. As the number of cores increases, routing algorithms must maintain efficiency without a significant increase in computational complexity. Moreover, ensuring low latency and high throughput while dynamically adapting to faults and traffic conditions is critical for maintaining network performance. Additionally, avoiding network deadlocks and preventing starvation, where some packets are indefinitely delayed, are key concerns in NoC design.

Our proposed routing strategy addresses these challenges by introducing a novel adaptive routing methodology that leverages the TOPSIS decision-making framework. The approach ranks routing paths based on multiple criteria such as path length, congestion levels, and link density to select the most reliable and efficient route. A key innovation of this method is the introduction of a stress value parameter, which quantifies link congestion and aids in optimizing routing decisions. Unlike many existing fault-tolerant methods, our approach maintains a low hardware footprint, making it suitable for large-scale NoC implementations. The stress value is updated dynamically based on real-time network conditions using an event-driven method, ensuring rapid adaptation to traffic changes and faults. This capability enhances network reliability while improving throughput and reducing latency. Furthermore, the proposed method ensures efficient congestion management and improves load balancing, leading to a more stable and high-performing NoC architecture. The main contributions of the research are as follows.

- Introduction of a QoS-aware adaptive routing mechanism that leverages TOPSIS for dynamic path selection, ensuring improved fault tolerance and efficient load balancing in NoCs.
- Implementation of a novel stress value parameter that quantifies link density, aiding in congestion management and enhancing routing decisions for improved network performance.

This paper is organized as outlined below. The second section examines routing methods in NoC systems, emphasizing fault tolerance and reviewing the relevant literature. The third section delineates the proposed strategy and elucidates the algorithmic specifics of the new method. The objective is to introduce a fault-tolerant routing solution in NoC systems to enhance the quality-of-service components. The proposed method also aims to enhance fault tolerance and refine evaluation criteria. The fourth section presents the simulation of the suggested method and evaluates the outcomes produced from it. Ultimately, in the fifth section, following a comprehensive conclusion, recommendations for future endeavors and citations are provided.

## Previous works

Network-on-a-chip (NoC) has emerged as an economical communication interface for multi-core tiling chip processor architectures. Inter-core communication occurs via packet exchange. As the computing demands of applications escalate, the frequency of packet transmission between cores correspondingly rises. Inadequate routing of these packets results in significant congestion, hence diminishing system performance^22^. This signifies the necessity for congestion-aware routing in NoC. In practical scenarios, apps operating on NoC produce varied traffic, hence posing routing issues. These issues have prompted an increasing number of researchers to depend on machine learning methods for resolution. Nonetheless, the challenges of storage overhead and packet latency prevail in these techniques. An adaptive routing algorithm, DeepNR, utilizing a deep reinforcement learning approach is introduced in^23^. The suggested methodology incorporates network data to depict the status, routing direction of activities, and queue delay for the reward function. Experiments including synthetic and contemporaneous traffic were performed to illustrate the efficacy and efficiency of DeepNR utilizing the Gem5 simulator.

In the field of network resilience, there have been works that explore “event-driven control” and “safety/security” approaches for distributed systems^24^: stable event-driven lossy interception with distributed delays; Ref^25^. present a fault-tolerant optimal scheme based on zero-sum game and dynamic adaptive programming; Ref^26^ investigate disturbance-resistant consensus with event-driven excitation and constraints; Ref^27^ present event-driven adaptive secure control for MAS with operator error and FDI attacks; Ref^28^ analyze observer-based secure control under DoS attacks in networked switching systems; and Ref^29^ advance fuzzy T–S based event-driven secure control against DoS.

A dynamic detection technique for wireless interface faults in WiNoC is proposed in^30^, which categorizes wireless interface error situations in WRs, executes wireless interface error detection during WiNoC operation, and revises the error scenarios. Additionally, an optimal path technique utilizing an error-free WR table is introduced to enhance network performance.

Various routes exist inside these networks to traverse from one node to another. Consequently, a function capable of identifying the optimal route to the destination should be accessible. The research^31^ employs an innovative hybrid approach termed Scored Regional Congestion Aware and DICA (ScRD) to optimize output channel selection and enhance the performance of NoC. Subsequent to the application of the ScRD algorithm, an analyzer scrutinizes the traffic packets to ascertain if the NoC communication is local or non-local, contingent upon the number of hops. Consequently, if the traffic is localized, the scoring process identifies the superior output channel. Alternatively, based on the system status and the specified parameter, the optimal output channel will be determined via the DICA or RCA selection functions. The Nirgam simulation was ultimately employed to evaluate the suggested approach across various traffic circumstances and selection criteria.

Reference^32^ introduces a fault-aware routing methodology tailored for mesh-based NoC architectures. The suggested method seeks to enhance the fault tolerance of NoC by strategically rerouting traffic to circumvent defective components while reducing performance deterioration. This method utilizes fault diagnosis mechanisms (BIST) and VC-based routing algorithms to effectively adjust to fluctuating network circumstances, ensuring resilient communications despite the presence of faults.

In^33^, a reinforcement learning-based fault-tolerant routing (RL-FTR) method is introduced to address routing issues arising from link and router failures in mesh-based NoC architecture. The efficacy of the proposed RL-FTR method is examined utilizing a System-C-based cycle-accurate NoC simulator. Simulations are conducted with an increasing number of links and router problems across various mesh sizes. Subsequent to the simulations, the real-time efficacy of the suggested RL-FTR method is evaluated by an FPGA implementation.

## Proposed system

The key aspect in developing a fault-tolerant routing algorithm is the quality-of-service parameters taken into account to attain the shortest pathways. Typically, fault-tolerant algorithms employ restricted criteria to identify a dependable path. This work proposes an adaptive routing system that identifies the most reliable way by assessing the status of surrounding nodes and integrating it with the path length. The suggested method employs the TOPSIS multi-criteria decision-making approach to rank pathways according to quality-of-service metrics. This algorithm originates from a Serbian term signifying a solution for compromise and multi-criteria optimization, initially developed in^31^. Upon the occurrence of a failure on a path, the algorithm identifies a substitute with comparable QoS attributes to transmit the packet, hence preserving efficiency throughout the failure and averting deadlock within the network.

### Defining the issue

**Fig. 1:**
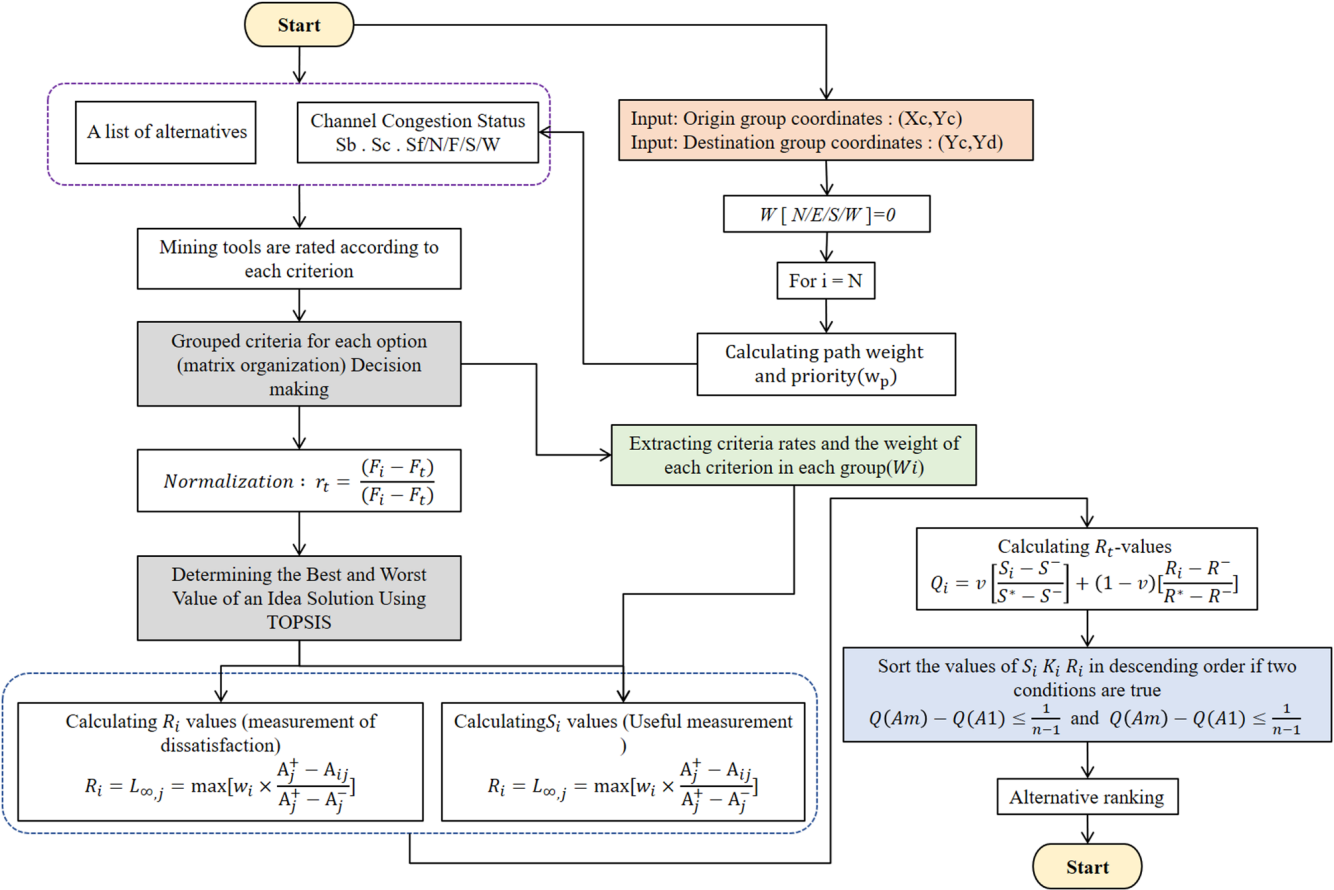
The suggested flowchart for TOPSIS.

Multi-criteria decision making (MCDM) or multi-criteria decision analysis (MCDA) is a branch of operations research that systematically assesses various conflicting criteria in the decision-making process. Effectively addressing intricate issues by explicitly evaluating several factors results in superior and more informed decision-making. The multi-criteria decision problem is articulated as follows: Identifying the optimal solution from a collection of viable alternatives assessed against a set of criterion functions. This paper introduces the comprehension of quality-of-service characteristics, the TOPSIS multi-criteria decision-making methodology, and its application for routing in NoC systems. Figure 1 illustrates the flow chart of the suggested method utilizing the TOPSIS algorithm.

*Step 1* Decision-matrix construction and raw feature extraction. Let $\:A=\{{a}_{1},\dots\:,{a}_{m}\}$ be the set of admissible next-hop routes from the current router $\:{v}_{c}=\left({x}_{c},{y}_{c}\right)$ toward the destination $\:{v}_{d}=\left({x}_{d},{y}_{d}\right)$. In a 2-D mesh, $\:A$ contains up to four output ports $\:\{N,E,S,W\}$ that keep the packet progress admissible (deadlock-free). For each alternative $\:{a}_{i}$ we compute three QoS criteria (two costs, one benefit) and assemble the $\:m\times\:n$ decision matrix $\:X=\left[{x}_{ij}\right]$ with $\:n=3$:

- $\:{C}_{1}$: remaining hop distance (cost). After selecting $\:{a}_{i}$, let the next node be $\:{n}_{i}=\left({x}_{i}^{{\prime\:}},{y}_{i}^{{\prime\:}}\right)$. The residual Manhattan distance is $\:{H}_{i}=\:\left|{x}_{d}-{x}_{i}^{{\prime\:}}\right|+\left|{y}_{d}-{y}_{i}^{{\prime\:}}\right|$. Set $\:{x}_{i1}={H}_{i}$.
- $\:{C}_{2}$: congestion/stress (cost). Each output port exposes a normalized stress $\:{S}_{i}\in\:\left[\text{0,1}\right]$ derived from neighbor input-buffer occupancy (EWMA) or its 3-level quantization (Low/Moderate/Severe). Set $\:{x}_{i2}={S}_{i}$.
- $\:{C}_{3}$: fault/health (benefit). Let $\:{h}_{i}\in\:\left[\text{0,1}\right]$ be the instantaneous health score of the link/next router on $\:{a}_{i}$ (1 = healthy, 0 = faulty), obtained from online detectors (parity/CRC, link-error counters) and periodic BIST. Set $\:{x}_{i3}={h}_{i}$.

Thus,

1 $$\:X=\:\left[\begin{array}{ccc}{X}_{11}&\:\cdots\:&\:{X}_{1i}\\\:⋮&\:\ddots\:&\:⋮\\\:{X}_{m1}&\:\cdots\:&\:{X}_{mJ}\end{array}\right]=\:\left[\begin{array}{ccc}{H}_{1}&\:{S}_{1}&\:{h}_{1}\\\:⋮&\:\ddots\:&\:⋮\\\:{H}_{m}&\:{S}_{m}&\:{h}_{m}\end{array}\right]$$

Costs ($\:{H}_{i},{S}_{i}$​) are minimized and the benefit $\:{h}_{i}$ is maximized. The weight vector $\:w=\left[{w}_{path},{w}_{cong},{w}_{fault}\right]\left(normalized\:to\:\sum\:{w}_{j}=1\right)$ is applied after normalization in Step 2. This Step defines the alternatives, the exact raw measurements, and the sign (cost/benefit) of each criterion; normalization and ideal points follow in Steps 2–3.

The decision matrix $\:\text{D}\in\:{\mathbb{R}}^{m\times\:n}$ contains the raw scores of the options; each row (i) represents a candidate path/port (west, south, east, north, or equivalent minimum-step paths), and each column (j) represents a QoS criterion (path length in hops, neighbor stress/traffic, health/fault). The entry $\:{D}_{ij}$ is extracted from local/neighbor counters at the decision moment. Column normalization of D per Eq. (2) yields the matrix A, which serves as the basis for calculating $\:{A}^{+},\:{A}^{-}\:$followed by $\:{S}_{i},\:{R}_{i}$, and $\:{Q}_{i}$​.

*Step 2* Normalization or scale-freeing constitutes the second phase in addressing all multi-criteria decision-making methodologies reliant on the decision matrix^33^. Normalization is conducted in a linear fashion as Eq. (2).

2 $$\:{A}_{ij\:\:}=\frac{{X}_{ij}}{\sqrt{\sum\:_{i=1}^{m}{X}_{i,j}^{2}}}$$

In this formula, $\:{X}_{ij}$ represents the j criterion for the $\:i-th$ path, and $\:m$ denotes the total number of pathways between the source and destination nodes. “Link-based transfer” with an efficient computational formulation for large-scale networks, which is related to the proposed discussion on scalability and time budget of link-based decision making^34^. The total of all elements post-normalization will equal one. Following normalization, if a negative criterion exists, its value must be derived Eq. (3).

3 $$\:{A}_{ij}=1-{A}_{ij}$$

Consequently, the standard decision matrix $\:A$ is derived Eq. (4). In this matrix^35^, $\:{A}_{ij}$ represents the normalized value of criteria j for the $\:i-th$ path between the source and destination nodes.

4 $$\:\left[\begin{array}{ccc}{A}_{11}&\:\cdots\:&\:{A}_{1i}\\\:⋮&\:\ddots\:&\:⋮\\\:{A}_{j1}&\:\cdots\:&\:{A}_{ij}\end{array}\right]$$

*Step 3* The third step involves identifying the optimal and suboptimal values for each criterion in the matrix. For positive criteria associated with profit, the highest value represents the best outcome, while the lowest value signifies the worst result. In negative criteria related to cost, the minimal value represents the optimal outcome, whereas the maximal value signifies the least favorable response^36^. The optimal and suboptimal values of each criterion are denoted as $\:{A}^{+}$ and $\:{A}^{-}$, respectively. The calculation of these two values employs (5) and (6).

5 $${A}^{+}=\text{max}{X}_{ij\:\:\:\:\:\:\:\:}$$

6 $${A}^{-}=\text{min}{X}_{ij}$$

In this context, $\:{A}^{+}$denotes the positive ideal, while $\:{A}^{-}$ signifies the negative ideal. Consequently, in this phase, the maximum and minimum values of each column in the decision matrix are identified^37^.

*Step 4* The fourth step involves calculating the utility value $\:{S}_{i}$ and the dissatisfaction value $\:{R}_{i}\:$ for the *ith* path between the origin and destination nodes. The utility value $\:S$ denotes the relative distance of the ith path from the ideal point, while the dissatisfaction value $\:R$ represents the greatest discontent of this road attributable to its distance from the ideal point^38^. To compute these values, Eqs. (7) and (8) are employed, respectively^39^.

7 $${S}_{i}={{L}_{1}}_{,j}=\sum\:_{j=1}^{n}{w}_{j}\times\:\frac{{A}_{j}^{+}-{A}_{ij}}{{A}^{+}-{A}_{j}^{-}}$$

8 $$\:{R}_{i}={{L}_{\infty\:}}_{,j}=\text{max}[{w}_{i}\times\:\frac{{A}_{j}^{+}-{A}_{ij}}{{A}_{j}^{+}-{A}_{j}^{-}}]$$

In this context, $\:{A}_{j}^{+}$denotes the optimal value for the *j*th criterion, $\:{A}_{j}^{-}$ signifies the suboptimal value for the jth criterion, $\:{w}_{i}\:$indicates the weight of the criterion^40^, reflecting its significance, and $\:{A}_{ij}$ represents the normalized value of criterion *j* for each path between the source and destination nodes within the conventional decision matrix.

*Step 5* In this phase, the computation of the TOPSIS index $\:{Q}_{i}$ for the *i*th path is executed. In this phase, the TOPSIS index value is computed using (9) for each path connecting the source and destination nodes.

9 $$\:{Q}_{i}=v\left[\frac{{S}_{i}-{S}^{-}}{{S}^{*}-{S}^{-}}\right]+\left(1-v\right)\left[\frac{{R}_{i}-{R}^{-}}{{R}^{*}-{R}^{-}}\right]$$

Scalable multi-agent routing with RL is presented as an alternative learning approach, while TOPSIS with guaranteed cyclic delay^41^. In this regard, the values of $\:{S}^{*}$, $\:{R}^{*}$, $\:{S}^{-}$and $\:{R}^{-}$ are calculated using (10) to (13) respectively, and the value of $\:v$ represents the weight of the strategy $\:{S}_{j}$, $\:{R}_{j}$ and $\:0<v<1$.

10 $$\:{S}^{*}=\text{max}{S}_{i}$$

11 $$\:{S}^{-}=\text{min}{S}_{i}$$

12 $$\:{R}^{*}=\text{max}{R}_{i}$$

13 $$\:{R}^{-}=\text{min}{R}_{i}$$

The parameter $\:v$ is also influenced by the consensus of the decision-making group. If the agreement is substantial, then $\:v>0.5$, if the agreement is determined by majority vote, then $\:v=0.5$ and if the agreement is minimal, then $\:v<0.5$ As $\:v$ increases, group viewpoints are prioritized, and as $\:v$ decreases, individual opinions are emphasized.

*Step 6* Sorting the routes based on the values of $\:{S}_{i}$, $\:{R}_{i}$and $\:{Q}_{i}$ is the next step. In this step, all the routes are sorted based on the values obtained from the above relations in order to select the ideal route based on the predefined conditions. For this purpose, the routes are sorted in three groups from small to large based on the values of $\:S$, $\:R$ and $\:Q$. The best route is the route that has the highest rank in all three values of $\:S$, $\:R$ and $\:Q$.Otherwise, the best route is the route that has the smallest $\:Q$.

*Step 7* Taking into account the two criteria of acceptable benefit and acceptable stability, ⅰ) the optimal path is the one that exhibits the lowest values across all three indicators, and ⅱ) there must be a difference between the first path (a) and the second path (b) in Eq. (14).

14 $$\:{Q}_{b}-{Q}_{a}\ge\:\frac{1}{m-1}$$

Acceptable stability in decision-making signifies that the selected compromise option must optimize collective value while minimizing individual repercussions. Should the initial condition remain unfulfilled, the first and second alternatives are regarded as preferable options^42^. If the second requirement is unmet, the first choice, as per the $\:Q$ ranking, to the final option that fails to satisfy the second condition are preferable alternatives.

### TOPSIS routing algorithm

To implement the proposed method, a weighted path strategy has been used. The number of steps between the source and destination nodes, prioritized by minimal path, and the channel status weights (busy/congested/failed) are computed in real time to establish the path priority weight criterion $\:{W}_{path}$ using the Vickers decision matrix. deals with the low-cost orchestration of network function chains for low-latency applications where multi-criteria decision policies can optimize service paths to minimize latency^43^; this idea is consistent with TOPSIS path selection. In the area of hardware resilience^44^, applies adaptive reconfiguration for redundancy and service continuation after failure, which is similar to the adaptive rerouting logic in NoC. Also^45^, presents a lightweight approach to fault tolerance in circuits by encoding hybrid random numbers to contain bit-width growth, which is relevant to our discussion of low overhead and energy efficiency in routing; otherwise, the path priority weight is determined as per Table 1. Additional weight values to be computed encompass the path priority weight $\:{W}_{path}$, the channel congestion weight $\:{W}_{Cong}$, and the channel fault weight $\:{W}_{Fault}$. Given that the algorithm initially selects movement along the x-axis followed by the y-axis under identical traffic conditions, the four cardinal ports north, east, south, and west (W/S/E/N) in the source node are designated as $\:p{p}_{2},p{p}_{1},p{p}_{3}$ based on the number of steps necessary to transmit packets to the destination. Both directions with an equal number of steps to the destination node are regarded at the same level.

**Table 1:** Calculating the weight of route priority.

Location of the node	Route priority level (*N*/E/S/W)	Route priority weight ($\:{W}_{path}$)
*(Xd > Xc) & (Yd = Yc)*	$\:p{p}_{1},p{p}_{3},p{p}_{2},p{p}_{1}$	[ 1,3,2,1]
*(Xd > Xc) & (Yd < Yc)*	$\:p{p}_{2},p{p}_{2},p{p}_{1},p{p}_{3}$	[ 2,2,1,3]
*(Xd < Xc) & (Yd < Yc)*	$\:p{p}_{2},p{p}_{3},p{p}_{1},p{p}_{2}$	[ 2,3,1,2]
*(Xd < Xc) & (Yd < Yc)*	$\:p{p}_{2},p{p}_{1},p{p}_{3},p{p}_{1}$	[ 2,1,3,1]
*(Xd < Xc) & (Yd = Yc)*	$\:p{p}_{3},p{p}_{2},p{p}_{1},p{p}_{2}$	[ 3,2,1,2]
*(Xd < Xc) &(Yd > Yc)*	$\:p{p}_{2},p{p}_{2},p{p}_{3},p{p}_{1}$	[ 2,2,3,1]
*(Xd = Xc) & (Yd > Yc)*	$\:p{p}_{2},p{p}_{3},p{p}_{2},p{p}_{1}$	[ 2,3,2,1]
*(Xd > Xc) & (Yd > Yc)*	$\:p{p}_{2},p{p}_{3},p{p}_{3},p{p}_{1}$	[ 2,3,3,1]

$\:p{p}_{1}$/$\:\:p{p}_{2}$/$\:\:p{p}_{3}$ are the only “path priority classes” for each outgoing port in the router: $\:p{p}_{1}$ (Primary): The minimum XY-aligned direction to the destination (highest priority). $\:p{p}_{2}$ (Secondary-minimal): The alternative minimum direction in the other axis, when pp1 is equal or blocked. $\:p{p}_{3}$ (Fallback): The two remaining directions that are usually non-minimum/less direct and are only used in case of failure/heavy congestion.

In the decision matrix, these labels are mapped to the numerical weight of the path: $\:p{p}_{1}$→1, $\:p{p}_{2}$→2, $\:p{p}_{3}$→3 (smaller numbers are better).

At each node, four cardinal directions (West, South, East, North) are evaluated to prioritize potential pathways. The classifications based on the number of steps and channel states provide the quantitative scoring values of the decision matrix. In response to the significant congestion surrounding the faulty nodes, the proposed method incorporates a congestion control mechanism utilizing a multi-level congestion strategy^45^. Each router possesses a stress value database that compares the stress values for each output channel^46^. The associated stress value is produced by adjacent routers and signifies the quantity of packets in the routers’ input buffer^47,48^.

The “stress” parameter is a per-port scalar in the range [0,1] that quantifies the congestion of the output path. It is updated using the normalized input buffer occupancy of the next-hop neighbor via EWMA: $\:{\text{s}}_{\text{t}}=\:={{\upalpha\:}}_{\text{o}\text{c}\text{c}\text{t}}\text{}+\left(1-{\upalpha\:}\right){\text{s}}_{\text{t}-1}$, with $\:{\upalpha\:}=0.2\:\left(\text{a}\text{n}\text{d}\:{\text{s}}_{0}=\:0\right)$. To prevent oscillation, hysteresis is applied: Low→Moderate when $\:{\text{s}}_{\text{t}}>\:0.47$ and Moderate→Severe when $\:{s}_{t}>\:0.87$ (reverts at < 0.80). each port every 8 cycles over the credit channel, and inputs expire after 64 cycles. This parameter directly serves as the congestion criterion in TOPSIS (either continuous or quantized to 3/5 levels).

Table 2 displays the anticipated congestion levels and corresponding stress values for each port. Three distinct congestion levels are delineated based on the degree of stress. Low congestion is defined as a state in which less than 47% of the buffer space is utilized, indicating a normal channel condition. The medium congestion level occurs when 47% to 87% of the buffer space is utilized, indicating a congested channel. Severe congestion occurs when over 87% of the buffer is utilized, designating the channel as congested.

**Table 2:** Density level calculation (stress value).

Channel status	Congestion level	Buffer occupancy percentage at neighboring node
Normal	Low	Less than 47%
Busy	Moderate	47% to 87%
Dense	Severe	Above 87%

The potential routes from the present node to the destination node extend in four directions: north, west, south, and east. Every node possesses a primary directional priority, $\:p{p}_{1}$, established using *XY* routing. Additionally, taking into account the number of steps to the target node, a medium-priority direction $\:p{p}_{2}$ and two low-priority directions $\:p{p}_{3}$ are taken into consideration. The weight of each level corresponds to its numerical index. For both paths with an equal number of steps to the destination node, the same level is taken into account. We shall elucidate the various stages of the proposed strategy by an illustrative case.

**Fig. 2:**
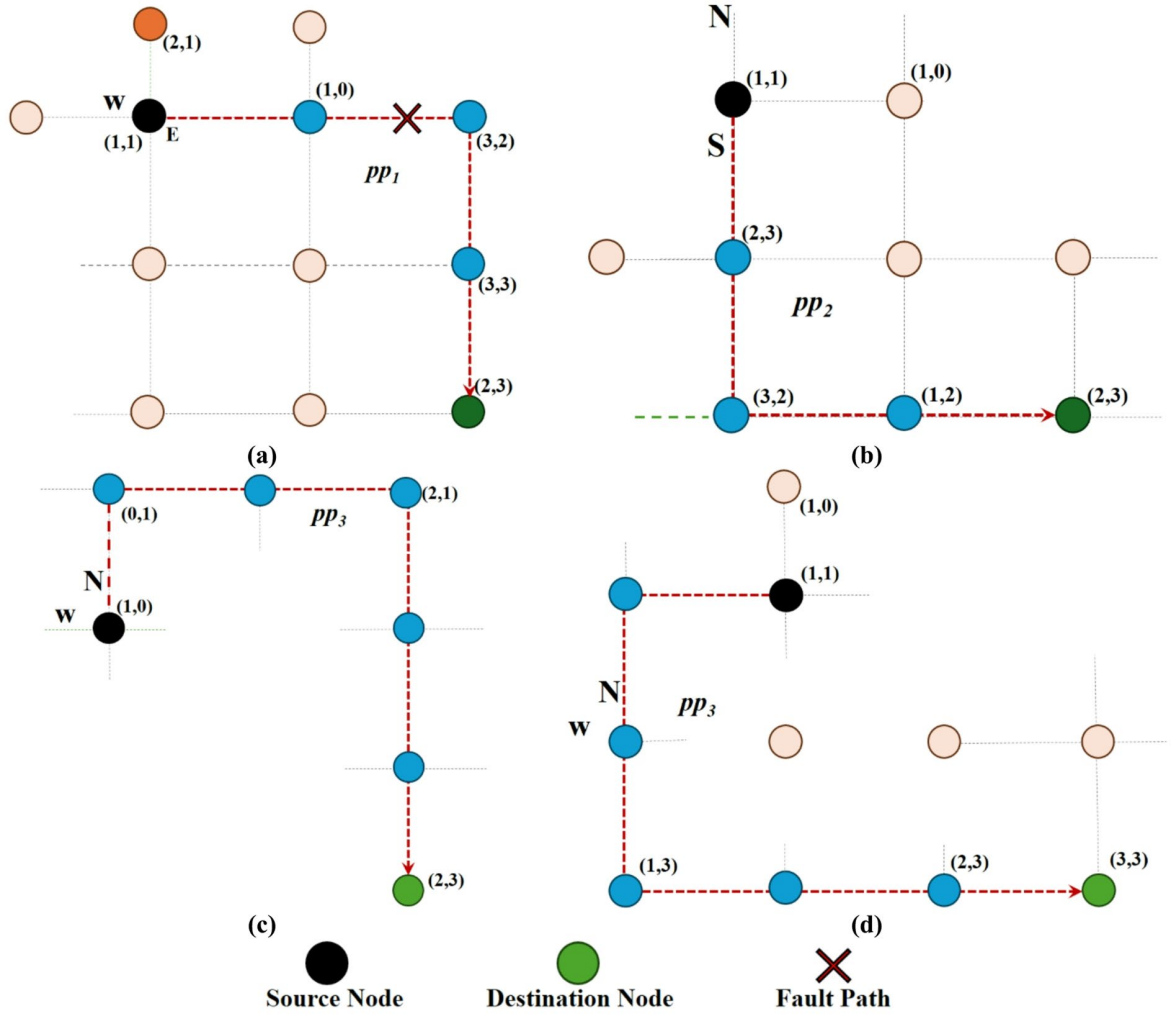
Various routes between the source and destination nodes according to the suggested approach. (**a**) Path of level port 1, (**b**) Path of level 1 port 2, (**c**,** d**) Path of level 3 port. (Color legend: blue = source router; orange = destination; light orange = intermediate routers on the highlighted $\:p{p}_{1}$/$\:\:p{p}_{2}$/$\:\:p{p}_{3}$ path.)

*Step 1* To enhance comprehension of the issue, examine the locations of the current and destination nodes as illustrated in Fig. 2. Node (1,1) serves as the current node, whereas node (3,3) represents the destination node. Consequently, path $\:p{p}_{1}$ is designated as the primary path due to its adherence to the *XY* routing pattern. Given that path $\:p{p}_{1}$ is likewise compromised, path $\:p{p}_{2}$ is designated as the secondary priority path with four steps, while $\:p{p}_{3}$ is assigned the lowest priority. The path priority weights and channel statuses (busy/congested/faulty) constitute the Vickers matrix for evaluation.

The path weight allocation method in Table 3 is as follows:

**Table 3:** Structure of the decision matrix for putting the suggested solution into practice.

Channel status	Neighbor node buffer size (fleet)	Path weight	Path priority	*N*
Busy	1	2	$\:p{p}_{2}$	E
Bad	Uncertain	1	$\:p{p}_{1}$	S
Normal	3	3	$\:p{p}_{3}$	W
Normal	4	2	$\:p{p}_{2}$	N

- *Step 1 (Manhattan Distance)*: The coordinate difference between the origin and destination at each node is calculated using the formulas $\:dx={X}_{d}-{X}_{c},\:dy\:=\:{Y}_{d}\--\:{Y}_{c}$ and the current distance $\:{D}_{0}=\left|{d}_{x}\right|+\left|{d}_{y}\right|$. A hypothetical step is taken and D′ (remaining distance) is calculated for each usable port (N/E/S/W).
- *Step 2 (Three-Level Ranking)*:
  - If the port’s movement is along the prioritized axis (in XY policy: x first, then y) and $\:{D}^{{\prime\:}}=D0-1\:\to\:\:pp1$ (highest).
  - If the movement is along the secondary axis and $\:{D}^{{\prime\:}}=D0-1\:\to\:\:pp2$ (medium).
  - If $\:{D}^{{\prime\:}}\ge\:{D}_{0}$​ (no improvement or worse) → pp3 (lowest). Ties are allowed; two ports may fall into the same level.
- *Step 3 (Mapping Rank to Numeric Weight)*: Ranks are mapped to cost weights: pp1 → 1, pp2 → 2, pp3 → 3 (smaller number = more desirable path). This corresponds to the “Path weight” column in Table 3.
- *Step 4 (Normalization for Decision Matrix)*: The values 1/2/3 are placed in the decision matrix and normalized (per Eq. (2)) to become scale-free, enabling comparison with other criteria (neighbor buffer size/channel status) in TOPSIS.

Figure 2 shows four candidate paths from origin (1,1) to destination (3,3) on a 4 × 4 mesh (coordinates are (x, y), x-axis pointing east and y-axis pointing north). In each subfigure, the red outline indicates the path being evaluated; the black circle is the origin, the green circle is the destination, the red × is the faulty link/port, the blue dots are the routers on the chosen path, and the light orange dots are the routers off the path. (a) Path pp1 follows the XY policy from east then north but is terminated due to an error in a final step (×). (b) Path pp2 bypasses the failure by changing the priority of the axes (first north then east) and remains minimal. (c) and (d) are examples of pp3 that take one or more initial non-minimal steps to avoid the faulty/congested area and then return to the minimal corridor. These four paths are the options that are ranked in the TOPSIS decision matrix; the labels pp1/pp2/pp3 next to each path indicate the path priority level according to Tables 1 and 3.

The northern port, characterized by path priority and the largest available buffer size, is optimally situated, whereas the eastern port, marked by the highest congestion, is in a congested location. *step 2*: To standardize the path priority weight and channel status, we shall have (2)^49^.

15 $$\:Ai{j}_{Weight}\left(N\right)=\frac{3}{3+2+3}=0.38$$

16 $$\:Ai{j}_{Weight}\left(W\right)=\frac{3}{3+2+3}=0.38$$

17 $$\:Aij\left(S\right)=\frac{2}{3+2+3}=0.25$$

Normalization of nearby node buffer sizes or channel state weights.

18 $$\:Ai{j}_{Buffer}\left(N\right)=\frac{1}{1+3+4}=0.13$$

19 $$\:Ai{j}_{Buffer}\left(W\right)=\frac{4}{1+3+4}=0.50$$

20 $$\:Ai{j}_{Buffer}\left(S\right)=\frac{3}{1+2+4}=0.37$$

To normalize negative criteria by subtracting from unity, based on (3), we have:

21 $$\:ri{j}_{Weight}\left(N\right)=1-0.36=0.64$$

22 $$\:ri{j}_{Weight}\left(W\right)=1-0.37=0.63$$

23 $$\:ri{j}_{Weight}\left(S\right)=1-0.24=0.76$$

The channel state post-normalization is presented in Table 4.

**Table 4:** Channel status after normalization.

Normalized buffer size	Normalization path priority weight	Possible routes
0.14	0.63	N
0.40	0.76	S
0.49	0.63	W

*step 3* The positive ideal represents the greatest value of each column^50^, while the negative ideal denotes the least value inside that column, as derived from (5) and presented in Table 5.

**Table 5:** Positive and negative ideal values.

Normalization	Positive ideal $\:{A}_{j}^{+}$	Negative ideal $\:{A}_{j}^{-}$
Route priority weight	0.78	0.64
Buffer size	0.56	0.15

*step 4* The suggested method assigns an identical weight influence coefficient $\:{W}_{i}$ of 0.44 to the three criteria: path priority weight, channel priority weight, and failure weight. The utility of the *i-th* path from the ideal point, denoted as $\:{S}_{i}$, and the unhappiness with the *i-th* path due to its deviation from the ideal value, denoted as $\:{R}_{i}$, are illustrated using Eqs. (7) and (8) and are summarized in Table 6. Determining the utility value $\:{S}_{i}$ and discontent $\:{R}_{i}$ for the northern gate^51^.

24 $$\:{S}_{i\left(N\right)}=\frac{0.44\times\:(0.76-0.63)}{0.76-0.63}+\frac{0.33\times\:(0.49-0.14)}{0.49-0.14}=0.44+0.44+0.77$$

25 $$\:{\:\:\:\:\:\:\:\:R}_{i\left(N\right)}=\text{max}\left(a.b\right)=0.44$$

**Table 6:** Utility value $\:{S}_{i}$ and dissatisfaction $\:{R}_{i}$ for each port.

Possible routes	$\:{S}_{i}$	$\:{R}_{i}$
N	0.77	0.44
S	0.22	0.22
W	0.44	0.44

Correspondingly, the criteria for the southern and western gates are established as follows:

26 $$\:\:\:\:\:\:\:\:{S}_{i\left(s\right)}=\frac{0.44\times\:(0.76-0.63)}{0.76-0.63}+\frac{0.44\times\:(0.49-0.39)}{0.49-0.14}=0+0.22=0.22$$

27 $$\:\:\:\:\:\:\:\:{R}_{i\left(s\right)}=0.22$$

28 $$\:\:\:\:\:\:\:{S}_{i\left(w\right)}=\frac{0.44\times\:(0.76-0.63)}{0.76-0.63}+\frac{0.44\times\:(0.49-0.49)}{0.49-0.14}=0.33+0=0.33$$

29 $$\:\:\:\:\:\:{R}_{i\left(W\right)}=0.44$$

Therefore, based on 10 to 13, we will have:

30 $$\:{S}^{*}=0.77$$

31 $$\:\:{S}^{-}=0.22$$

32 $$\:\:{R}^{*}=0.44$$

33 $$\:\:{R}^{-}=0.22$$

*Step 5* The $\:{Q}_{i}$ index is calculated from (9) and the results are shown in Table 7.

**Table 7:** Possible paths after determining the TOPSIS index.

Possible routes	$\:{S}_{i}$	$\:{R}_{i}$	$\:{Q}_{i}$
N	0.77	0.44	1
S	0.22	0.22	0
W	0.44	0.44	0.7
N	0.77	0.44	1

34 $$\:\:{Q}_{I\left(N\right)}=0.6\left[\frac{0.77-0.22}{0.77-0.22}\right]+\left(1-0.6\right)\left[\frac{0.44-0.22}{0.44-0.22}\right]=1$$

35 $$\:\:{Q}_{i\left(S\right)}=0.6\left[\frac{0.22-0.22}{0.77-0.22}\right]+\left(1-0.6\right)\left[\frac{0.22-0.22}{0.44-0.22}\right]=2$$

36 $$\:{Q}_{I\left(W\right)}=0.6\left[\frac{0.44-0.22}{0.77-0.22}\right]+\left(1-0.6\right)\left[\frac{0.44-0.22}{0.44-0.22}\right]=0.8$$

*Step 6* The southern gateway $\:S$ possesses the lowest value across all three indices and is thus designated as the optimal route based on the initial criterion. As all three routes require ranking, the western route $\:W$, possessing a $\:{Q}_{i}$ index lower than that of the northern route $\:N$, is consequently ranked first, followed by the northern route in second place. The findings are displayed in Table 8.

**Table 8:** Ranking of possible paths.

Possible routes	The best route	First place	Second place
N			$\:\surd\:$
S	$\:\surd\:$		
W		$\:\surd\:$	

Algorithm1 illustrates the pseudocode of the suggested algorithm utilizing the TOPSIS methodology. In fault-free NoC, the proposed solution is expected to follow a longer, unobstructed path. In regions characterized by convex defects^49^, packets are initially directed along the network’s perimeter before proceeding towards the region’s corner. This circumstance is associated with the initiation of the rerouting condition mechanism for areas with concave faults^15^ or other critical situations. If the number of rerouting surpasses the threshold, the router discards packets by restricting the rerouting count. The consequence of packet dropping is the preservation of system traffic load equilibrium by diminishing communication links and averting deviation. Upon packet loss, the faulty port is deactivated by the^52^ method prior to retransmission, followed by the implementation of the automated retransmission request (ARO) mechanism. The strategy can furthermore be employed to avert wandering in concave fault regions by transforming them into convex fault regions. However, even when opting for a non-minimum route in impaired and crowded regions, there is assurance of successful packet delivery.

**Algorithm 1:**
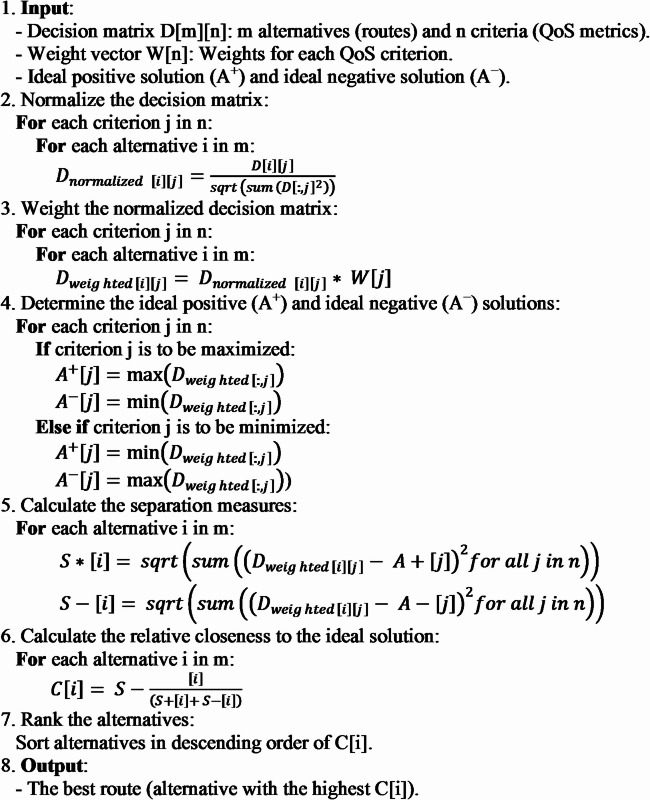
Pseudocode using the TOPSIS method.

Algorithm 1 in the paper presents a pseudo-program code of the TOPSIS multi-criteria decision-making method, which is used in the routing framework in NoC. This algorithm, considering QoS criteria such as path weight, channel density, and failure status, first normalizes the decision matrix and then calculates the weighted normalized matrix by applying the specified weights. Next, the positive and negative ideal solutions for each specified criterion and the distance of each path from these solutions are measured. Finally, the best possible path is selected by calculating the relative proximity of each path to the ideal solution. This adaptive approach, while maintaining load balance and avoiding congestion or deviation, allows finding the optimal alternative path even under conditions of network failure or severe congestion.

### Energy modeling

To evaluate the energy efficiency of the proposed algorithm, a trace-based modeling approach is used using ORION 3.0, a well-known analytical framework for estimating the power of routers and links in NoC systems. The simulation environment mirrored that outlined in Sect. 4, utilizing traces gathered from the Noxim simulator. During each simulation run, the subsequent router and link activity were recorded:

Input buffer read and write operations, Virtual-channel allocator (VA) requests and grants, Switch allocator (SA) decisions, Crossbar traversals, and Link transmissions per flit.

The event counts were supplied to ORION 3.0, configured for a 45 nm technology node, with a supply voltage of 1.0 V, a clock frequency of 100 MHz, and a flit width of 36 bits. Each router was designed with 5 ports and 2 VCs per port, featuring a FIFO depth of 8 flits.

The total energy was calculated as:

37 $$\:{E}_{total}=\sum\:_{e}{N}_{e}\times\:{E}_{e}^{dyn}+{T}_{sim}\times\:{P}_{leak}$$

where $\:{N}_{e}$ denotes the quantity of occurrences classified as eee, $\:{E}_{e}^{dyn}$ represents the dynamic energy per event, $\:{P}_{leak}$ denotes the leakage power, and Tsim represents $\:{T}_{sim}\:$denotes the simulated execution duration. The average power and normalized energy measures were derived as follows:

38 $$\:{P}_{avg}=\frac{{E}_{total}}{{T}_{sim}},\:\:\:\:\:\:{E}_{flit}=\frac{{E}_{total}}{{N}_{flits}},\:\:\:\:\:\:\:{E}_{packet}=\frac{{E}_{total}}{{N}_{packet}}$$

where $\:{N}_{flits}$ and $\:{N}_{packet}$ denotes the quantity of successfully transmitted flits and packets, respectively. All algorithms (XY, DyAD, EDAR, and the proposed TOPSIS) were assessed using the identical Orion configuration to ensure fairness.

### Fault model and robustness to transient/environmental effects

Practical NoCs encounter transitory problems such soft upsets, timing mistakes caused by voltage droop, and thermal hotspots, while Sects. 3–4 assess permanent (static) link/router defects. Therefore, we expand the model to include environmental context and time-varying reliability.

process of transient faults. A two-state process $\:\left\{G,B\right\}$ (good/bad) is followed by each link/router $\:e$. With probability of p and r per cycle, transitions $\:G\:\to\:\:B$ and $\:B\to\:G$ occur, resulting in average bad-burst length $\:\frac{1}{r}$ and inter-burst length $\:\frac{1}{p}$. The port may throttle or momentarily disable during $\:B$, and the local error rate increases.

Health score (decayed with time). The health score of routers is $\:{h}_{e}\left(t\right)\in\:\left[\text{0,1}\right]$.Updated from event counters (CRC/NACKs, retran, parity) with exponential decay, he(t)∈[0,1] per outgoing port:

39 $$\:\left(t-1\right)+\left(1-\beta\:\right)\left[1-{err}_{norm}\left(t\right)\right],\:\:\:\:\:\:\:\beta\:\in\:\left(\text{0,1}\right)$$

awareness of voltage and temperature. Let $\:{k}_{V}{\delta\:}_{v}$ be a droop indicator (1 during identified droop windows) and $\:{T}_{e}$ be the local link/router temperature.

40 $$\:{A}_{e}^{fault}\left(t\right)=1-{h}_{e}\left(t\right)$$

In order to increase the influence of $\:{A}_{e}^{fault}$, we scale the fault weight as follows:

41 $$\:{w}_{e}^{fault}={w}_{0}[1+{k}_{T}\left({T}_{e}-{T}_{ref}\right)+{k}_{V}{\delta\:}_{v}]$$

in areas with droops or hotspots. On-chip sensors or a runtime estimator can provide the temperature; the power-integrity monitor can provide the droop windows.

### Error detection, delay, and coverage resources

Periodic BIST, link integrity monitors (error counters/NACKs), and light online data path checks (buffer balance, flit-level CRC) are used. Table 9 summarizes the relevant detection latency and assumed coverage for each fault class, which include structural faults covered by periodic BIST at 95–99%, link monitors observable in ≤ 2 cycles at online coverage of 90–95%, and parity/CRC flags in 1 cycle with single-bit coverage ≥ 99%.

**Table 9:** Detection sources, fault classes, latency, and assumed coverage.

Fault class	Detection source	Latency (cycles)	Coverage (assumed)	Notes
Single-bit data upset	Parity / CRC	1	≥ 99% (single-bit)	CRC flags at egress; parity in buffers
Multi-bit data upset	CRC (flit-level)	1	90–99%	Depends on CRC polynomial/width
Link intermittent (crosstalk, EM)	Link error counter	≤ 2	90–95% (online)	Counters thresholded with hysteresis
Router pipeline transient	Parity/CRC + retry	1–2	90–95%	Detected via retrans/timeout
Structural (stuck-at/open/short)	Periodic BIST	Test interval	95–99%	Run in idle/low-load windows

Aggregation budget and latency of the router. Link counter updates arrive in a sideband in ≤ 2 cycles, TOPSIS re-ranking is implemented in 1–2 cycles, and local parity/CRC tokens are utilized in 1 cycle. As a result, end-to-end routing usually takes 3 to 5 cycles (local) or 5 to 7 cycles (non-local), which is in line with the per-source delays mentioned in Table 10 and does not increase the data stream’s critical path.

**Table 10:** 8 × 8 mesh, transient/environmental scenarios (throughput in flits/cycle, delay in cycles).

Scenario	Algorithm	Throughput	Delay
Transient bursts	XY	3.12	61
	DyAD	3.78	53
	EDAR	4.86	45
	TOPSIS	5.02	41
Thermal gradient (ΔT ≈ 30 °C)	XY	3.35	58
	DyAD	3.91	51
	EDAR	4.79	46
	TOPSIS	4.96	42
Voltage droop (5% cycles)	XY	3.28	60
	DyAD	3.85	52
	EDAR	4.82	46
	TOPSIS	4.98	42

### Cycle-level latency and complexity

The complexity is $\:O(P\times\:C)\:(2D:\:P=4;\:3D:\:P=6;$ here $\:C=3$: path length, congestion/stress, error/health). The routing decision is entirely local and scales with the number of ports assessed (P) and criteria (C), not with the network size. The calculations are performed using prenormalized and preloaded inputs $\:\left({A}^{+},{A}^{-}\right)$ in a 16-bit fixed point (Q8.8). All that is needed for each port is addition/replacement, multiplication, and max/compare; roots are not needed for the ranking. The decision kernel is a pipeline with two stages: $\:{S}_{i}$ and $\:{R}_{i}$ are computed on the ports in parallel by S1; S2 creates $\:{Q}_{i}$ and chooses the best result. The kernel encounters the same frequency target as the base router because the status and health signals arrive in a sideband. In accordance with the per-source delays in 4.3, the end-to-end rerouting delay from error indication to next-hop update is around 3–5 cycles when issues are local to the router and approximately 5–7 cycles for non-local situations. When scaling from 4 × 4/8 × 8 to 16 × 16 or to 3D, the two-cycle decision cost stays the same because $\:P$ is modest and constant in any architecture.

## Simulation and evaluation of results

The algorithm assessment platform described in the research utilizes 2D grid aggregation, with simulations conducted via the Noxim simulator^28^ on a personal computer equipped with 16 GB RAM and an Intel^®^ CoreTM i3-7100 CPU operating at 512.4 GHz within a Linux environment. The proposed solution is assessed using the conventional metrics of throughput rate T and average delay D, derived from Eqs. (15) and (16) in references^19,32^.

42 $$\:\:T=\frac{{R}_{flist}}{{N}_{nodes}-{N}_{clk}}$$

Where in (42), $\:{N}_{nodes}$ is the number of nodes, $\:{R}_{Alist}$ is the total number of received fleets, and $\:{N}_{clk}$ is the number of clock cycles from the first fleet generated to the last fleet received. Equation (43) defines the average delay *D*, which is the average delay value for the total number of messages, where $\:k$ is the total number of messages reaching their destination nodes and $\:{D}_{i}$ is the delay for *i*.

43 $$\:D=\frac{1}{k}\sum\:_{i=1}^{k}{D}_{i}$$

To guarantee the precision of the results, the simulation was conducted five times at each packet injection rate (PIR), and the outcomes are reported as an average. The initiation and execution durations are 1000 and 10,000 clock cycles, respectively. The routing algorithm is assessed using many prevalent performances estimate models, including random traffic, shuffled traffic, and transpose traffic.

The graphs derived from the simulation under failure-free conditions of the proposed method compared to the congestion-aware technique^17^ EDAR and the two algorithms, XY and DyAD, are evaluated under identical traffic conditions. Due to the differing topologies of various systems, the results generated are adjusted by decreasing the throughput rate via the simulator. The distinction between the suggested solution and the EDAR algorithm is in the method of weighing the criteria. In EDAR, the failure condition, congestion condition, and busy condition significantly affect channel performance, in that order.

Upon applying a basic cumulative ranking method to assess the paths, EDAR computes the total of the indices for each alternative, with the option possessing the lowest total establishing the priority path. The proposed method, after optimizing the weight with the most significant status ($\:{W}_{Busy}/{W}_{Cong}/{W}_{Fault}$), focuses on ranking the paths. If an optimal and unique solution is achieved in the optimization of the first criterion function, the problem is resolved; otherwise, the optimization of the second criterion function will be conducted. The procedure persists in accordance with priority until the issue is fully resolved. The suggested approach evaluates each indicator independently from the others, establishing a framework for assessing competing possibilities, ensuring that the chosen path maximizes collective utility while minimizing individual impact.

### Results of efficiency

**Fig. 3:**
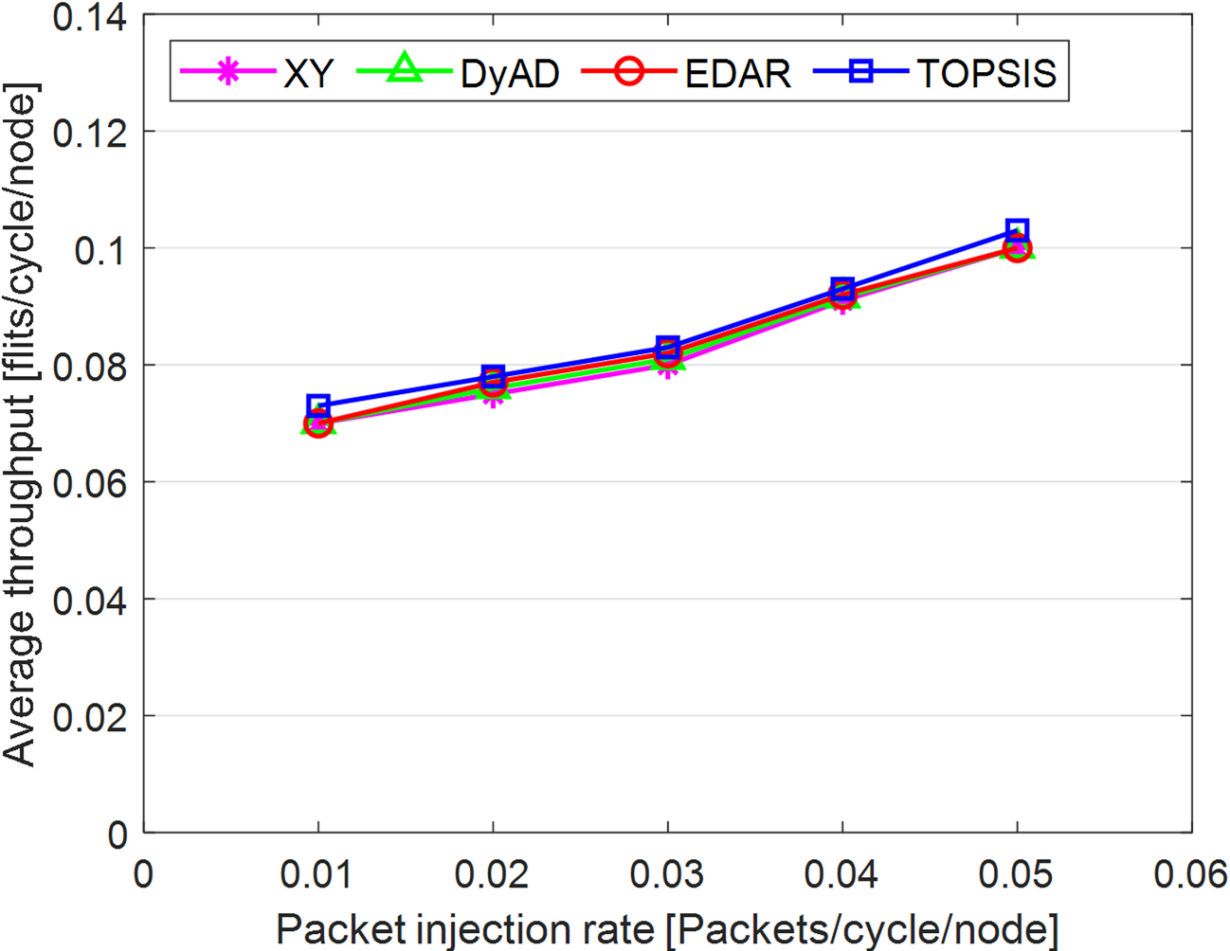
Average throughput rates of different routing algorithms under traffic load.

**Fig. 4:**
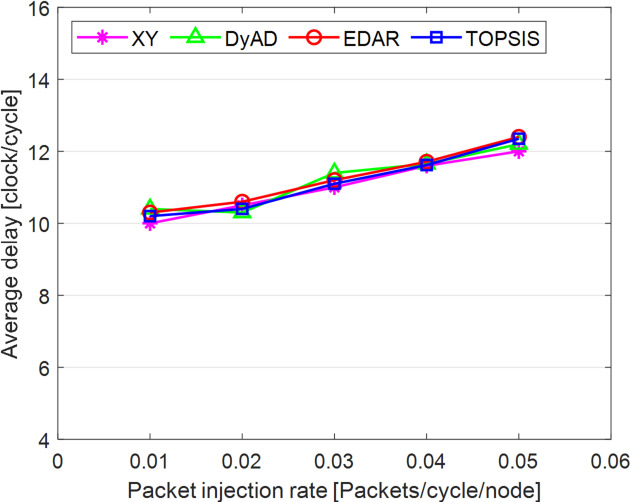
Average delay rate of different routing algorithms under traffic load.

**Fig. 5:**
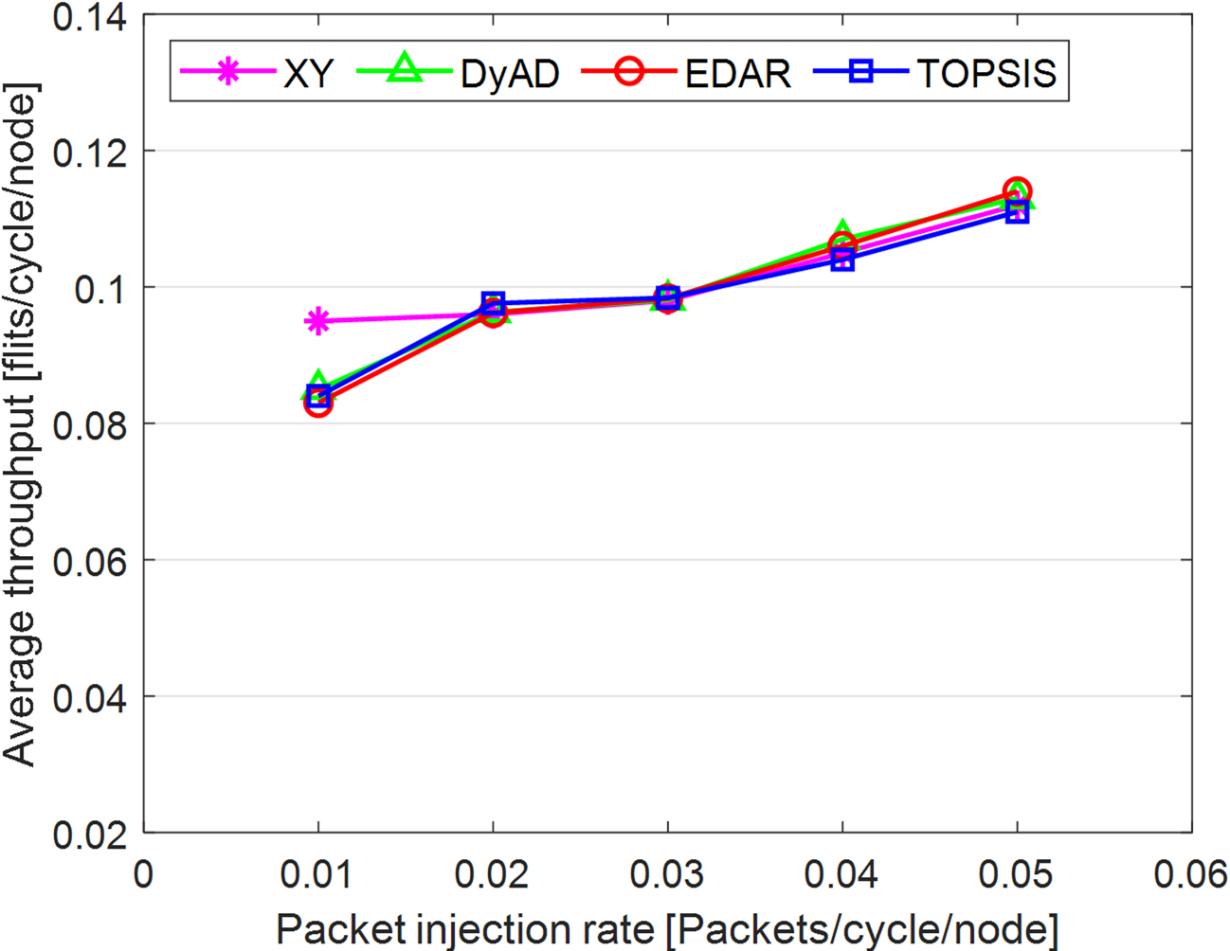
Average throughput rates of different routing algorithms under Shuffle load.

**Fig. 6:**
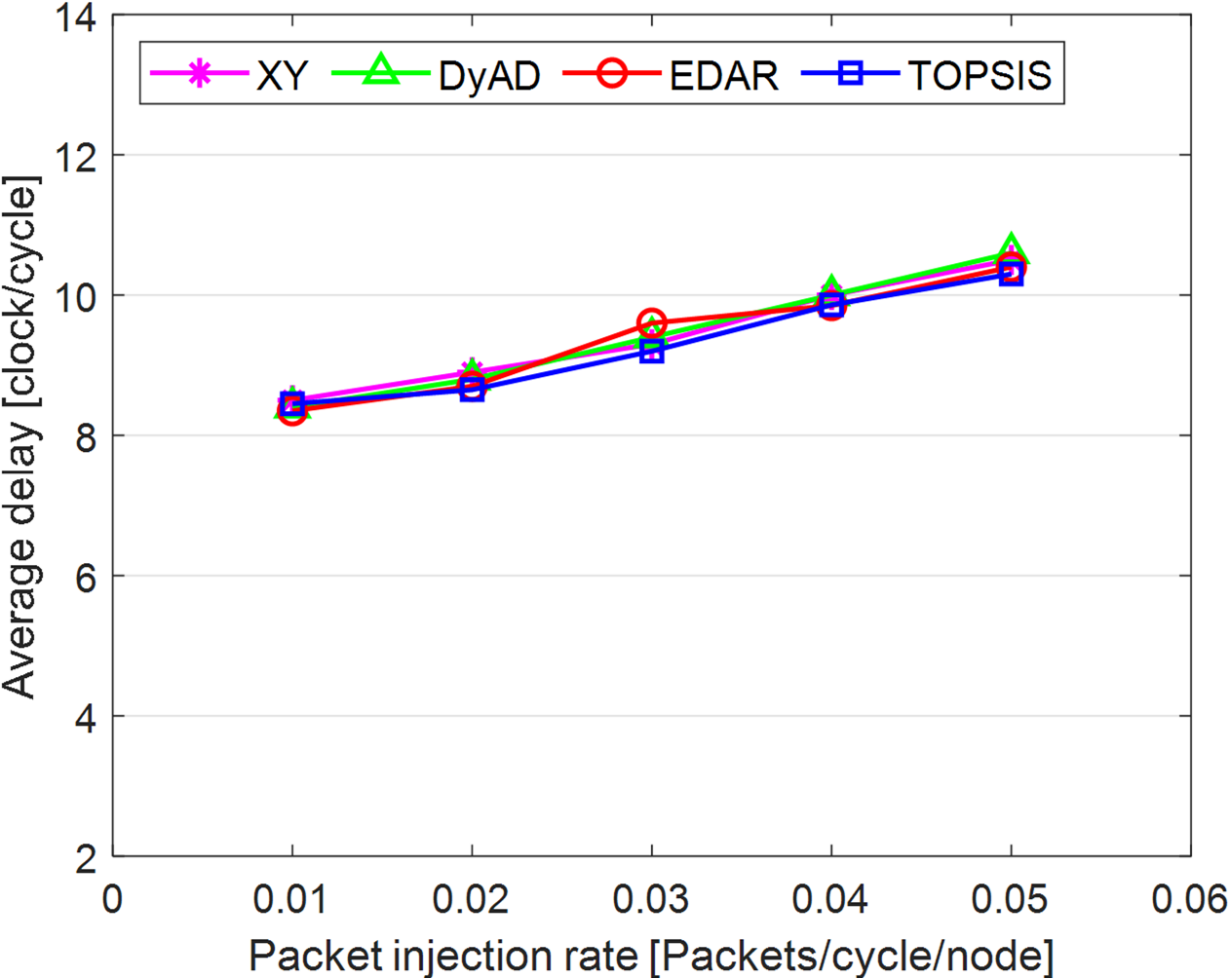
Average delay rate of different routing algorithms under Shuffle load.

**Fig. 7:**
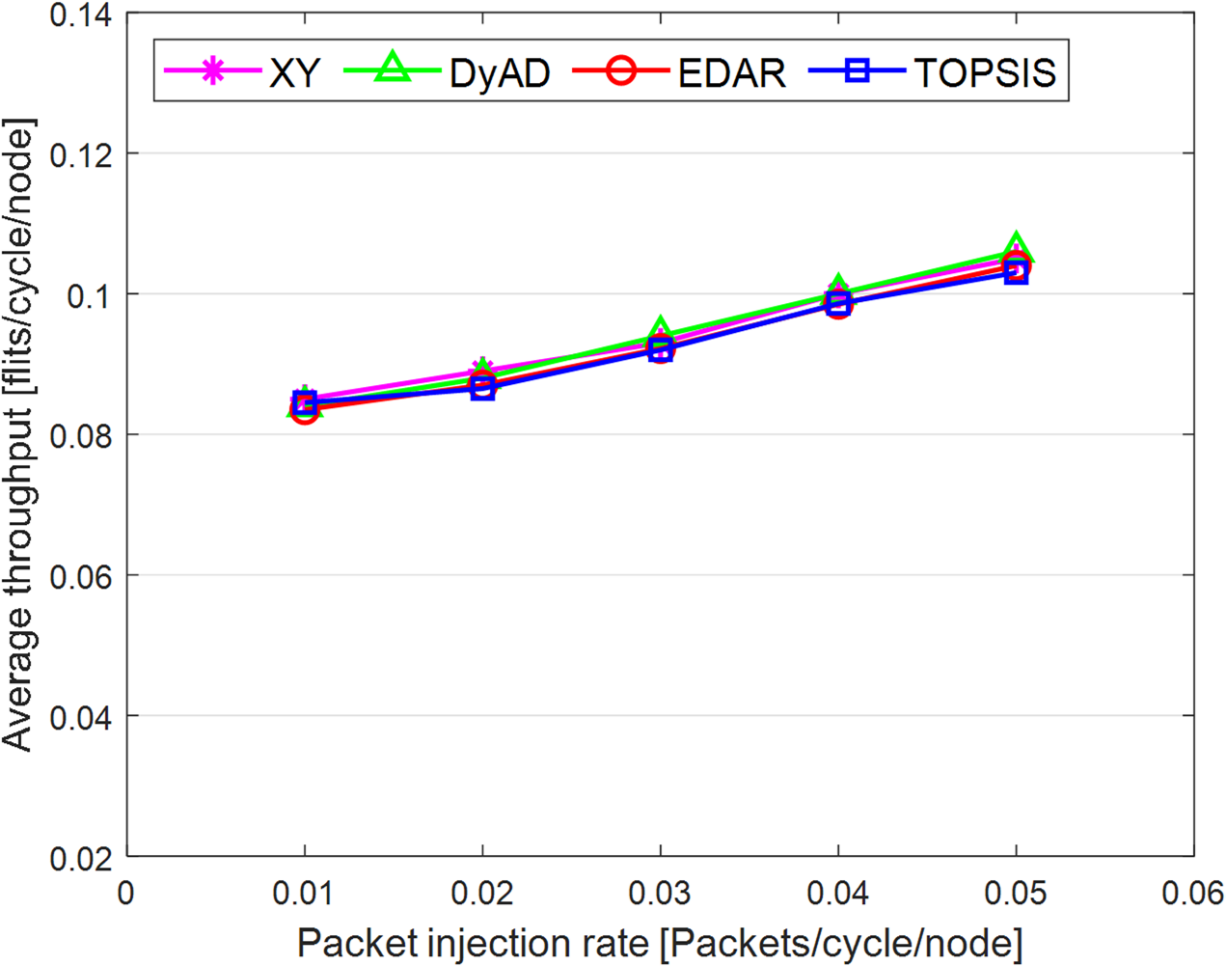
Average throughput rates of different routing algorithms under Transpose load.

**Fig. 8:**
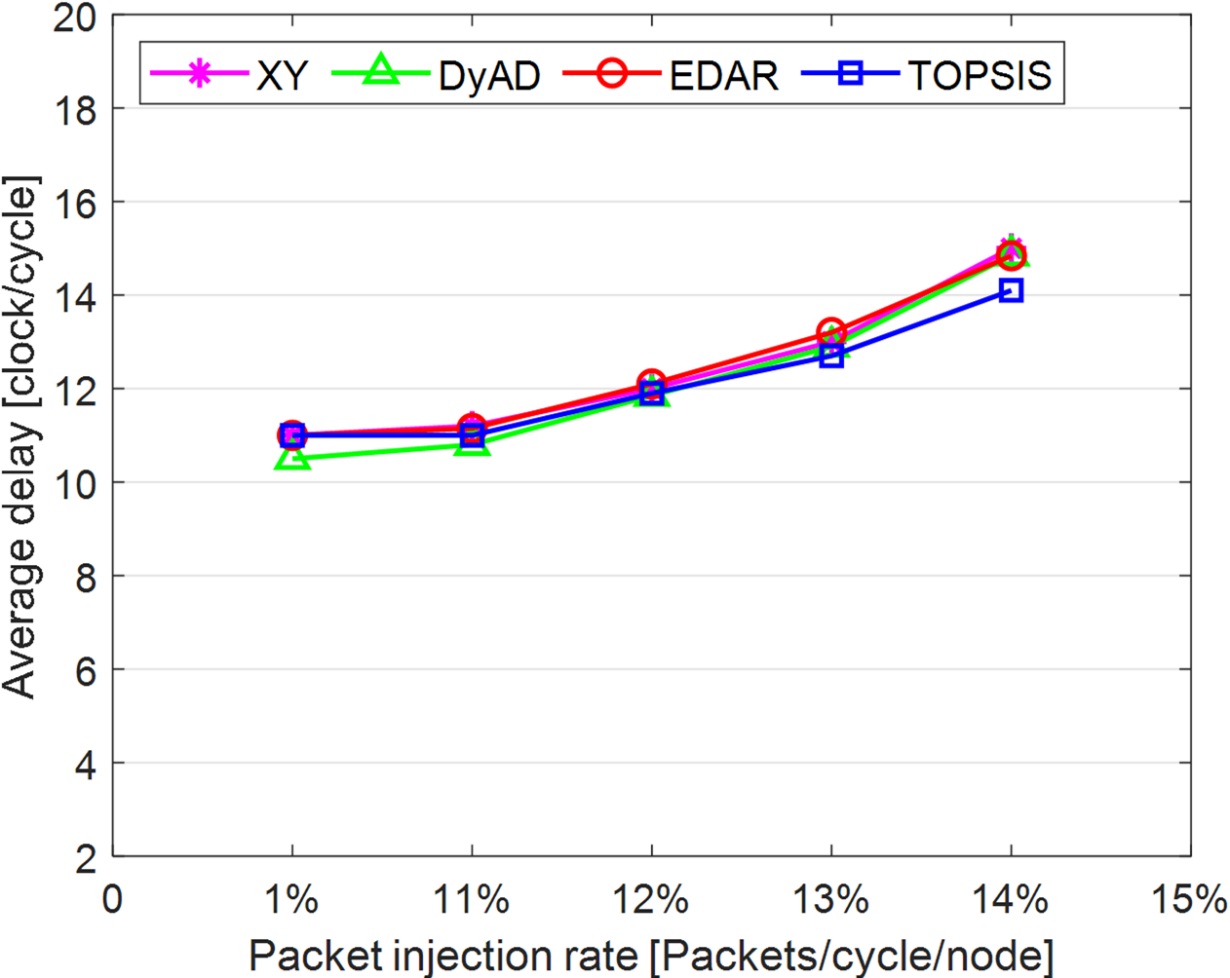
Average delay rate of different routing algorithms under Transpose load.

The TOPSIS router is simulated in 4 × 4 and 8 × 8 mesh networks under fault-free conditions, followed by scenarios with 5%, 10%, 15%, and 20% link failures, since all routing algorithms demonstrate efficiency with up to 20% link failure. Figures 3, 4, 5, 6, 7 and 8 illustrate the average throughput and delay rate under Random, Shuffle, and Transpose traffic loads in fault-free situations.

Under optimal conditions, the suggested routing algorithm demonstrates reduced average delay and enhanced throughput relative to the EDAR algorithm. The similarity of the experimental outcomes of the suggested approach to the XY and DyAD algorithms arises from the proposed algorithm’s adherence to the XY technique in fault-free conditions. The XY routing algorithm adjusts to the traffic flow over time. Additionally, if feasible, uniform traffic is established by directing packets initially along the X axis and subsequently along the Y axis. Conversely, adaptive routing algorithms temporarily reserve the chosen channels, as this decision-making may disrupt network traffic over an extended duration. Figure 3 illustrates that, under fault-free conditions, the throughput figures for all patterns exhibit uniformity up to a 5% packet injection rate when subjected to random traffic load. In Fig. 4, the delays stay nearly constant under same traffic load and packet injection rate. The elevated delay rates of EDAR and TOPSIS are attributable to their adaptive architecture. Adaptive algorithms incur a minor latency as a result of executing additional computations in fault-free situations. In Fig. 5, under identical settings as previously, with Shuffle traffic load, all four patterns exhibit consistent throughput rates. The TOPSIS model demonstrates increased throughput at a 1% closed injection rate, potentially attributable to the experimental conditions. Figure 6 demonstrates that, akin to the Random traffic load scenario, the delay rate of the patterns remains consistent under Shuffle traffic load.

All four algorithms practically converge to the (XY)-dimensional routes in Figs. 3, 4, 5 and 6, when the network is ideal and the injection load is in the pre-saturated area. In this regime, the efficiency is determined by the minimal path length and the queues stay small. In our model, the TOPSIS decision matrix reduces analytically to the “path length” criterion when the “failure/health” criterion equals 1 and the “stress/congestion” criterion is near zero. As a result, TOPSIS is purposefully designed to not impose any penalty (no power loss or delay increase) under healthy conditions, and the differences stay within the measurement noise limit. Because of topology symmetry, the Shuffle and Transpose patterns also activate the identical minimum routes; as a result, the power and delay curves for each approach line up. Crucially, the scientific objective at this time is to prove the “costlessness” of comparison logic in everyday situations.

In Fig. 7, the throughput rate stability of the four designs under Transpose traffic load resembles that of the XY pattern. The DyAD routing algorithm functions in a deterministic mode under low traffic conditions to minimize delay. Consequently, network efficiency improves during periods of both low and high congestion. The EDAR algorithm consistently executes routing adaptively, resulting in increased delay under non-congested conditions. Nonetheless, the suggested approach continues to function with reduced average delay and enhanced throughput compared to the adaptive EDAR pattern. Employing the packet injection rate within the saturation threshold as a reference value facilitates an equitable evaluation of system performance under uniform and faultless settings for all examined routing methods.

As the fault rate rises to 10%, the suggested approach experiences a 5% decline in average throughput, and at a fault rate of 20%, this decline is roughly 20%. Under this rate, XY will see an average throughput drop of 70%, whereas DyAD will encounter an average throughput reduction of 60%.

**Fig. 9:**
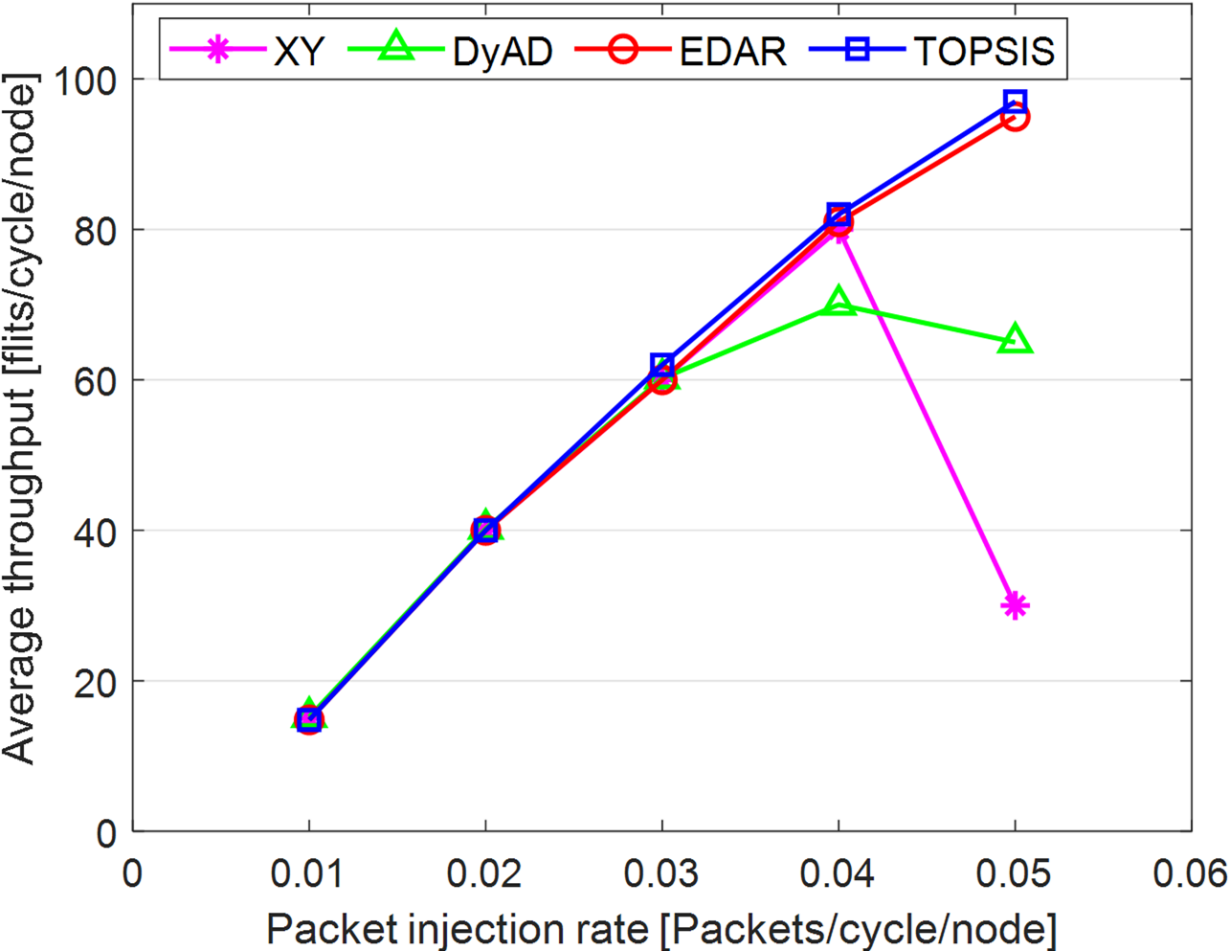
Evaluation of the average throughput rate under 5% to 20% failure conditions of different algorithms under random traffic load.

**Fig. 10:**
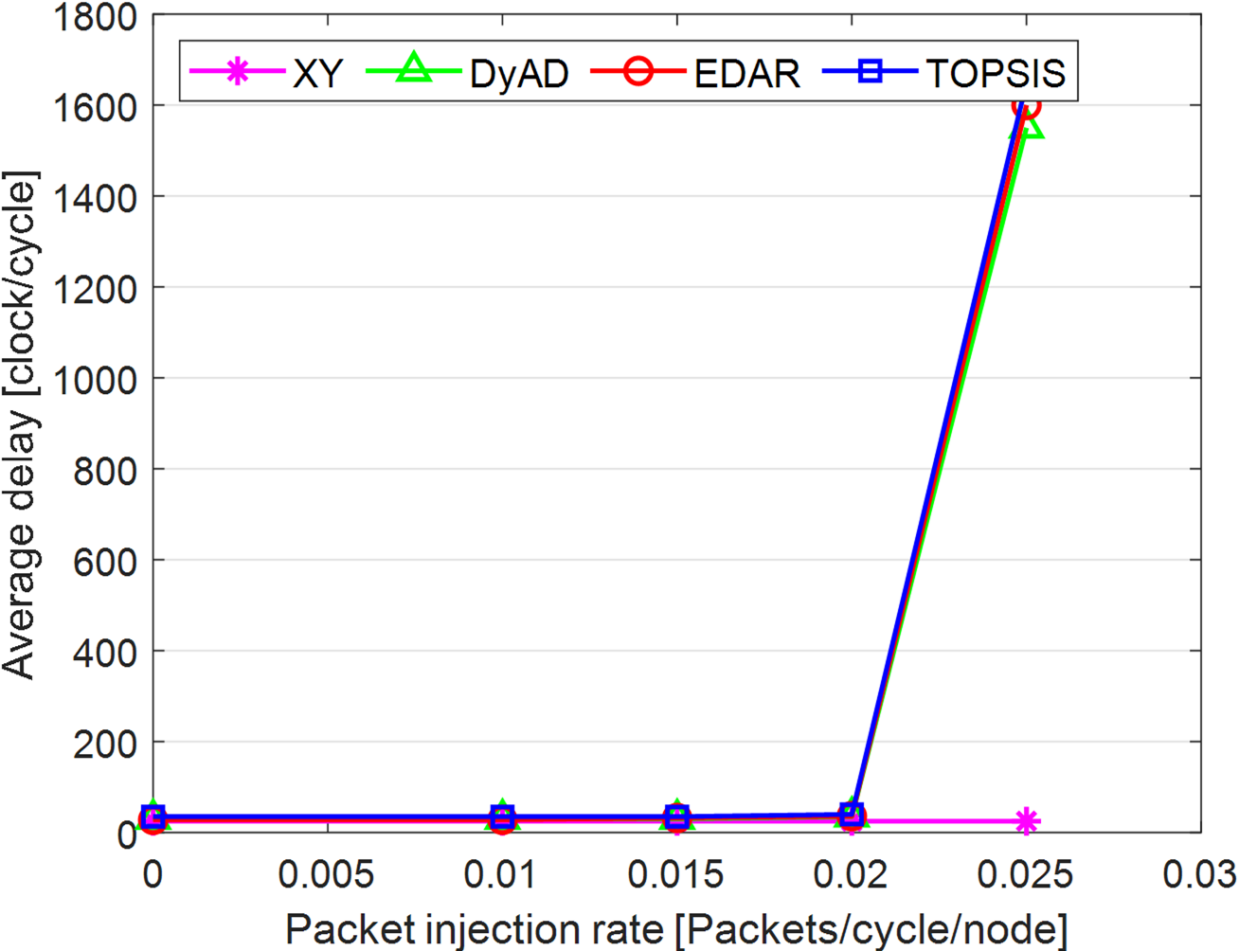
Evaluation of the average delay rate under failure conditions of 5% to 20% for different algorithms under random traffic load.

**Fig. 11:**
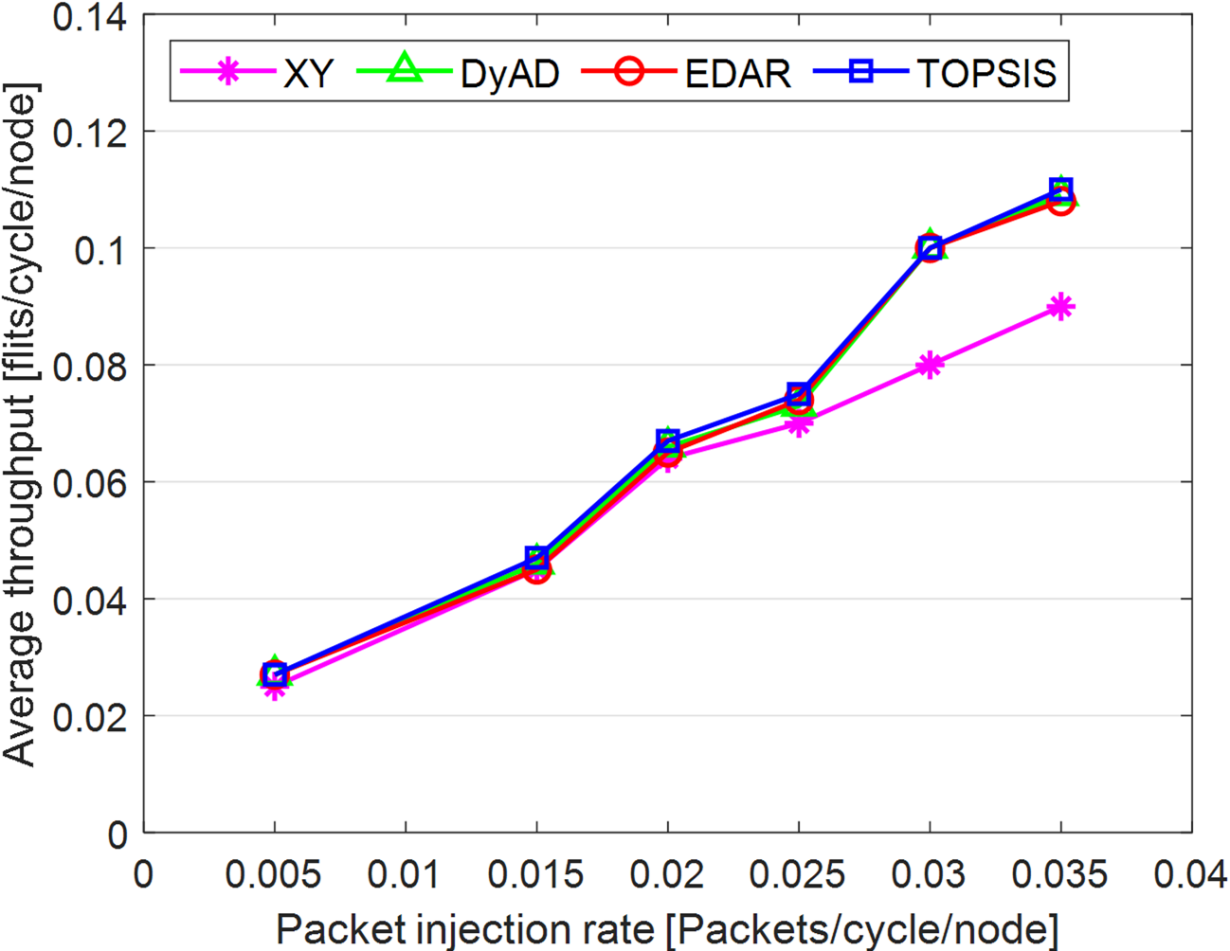
Evaluation of the average throughput rate under 5% to 20% failure conditions of different algorithms under traffic load.

**Fig. 12:**
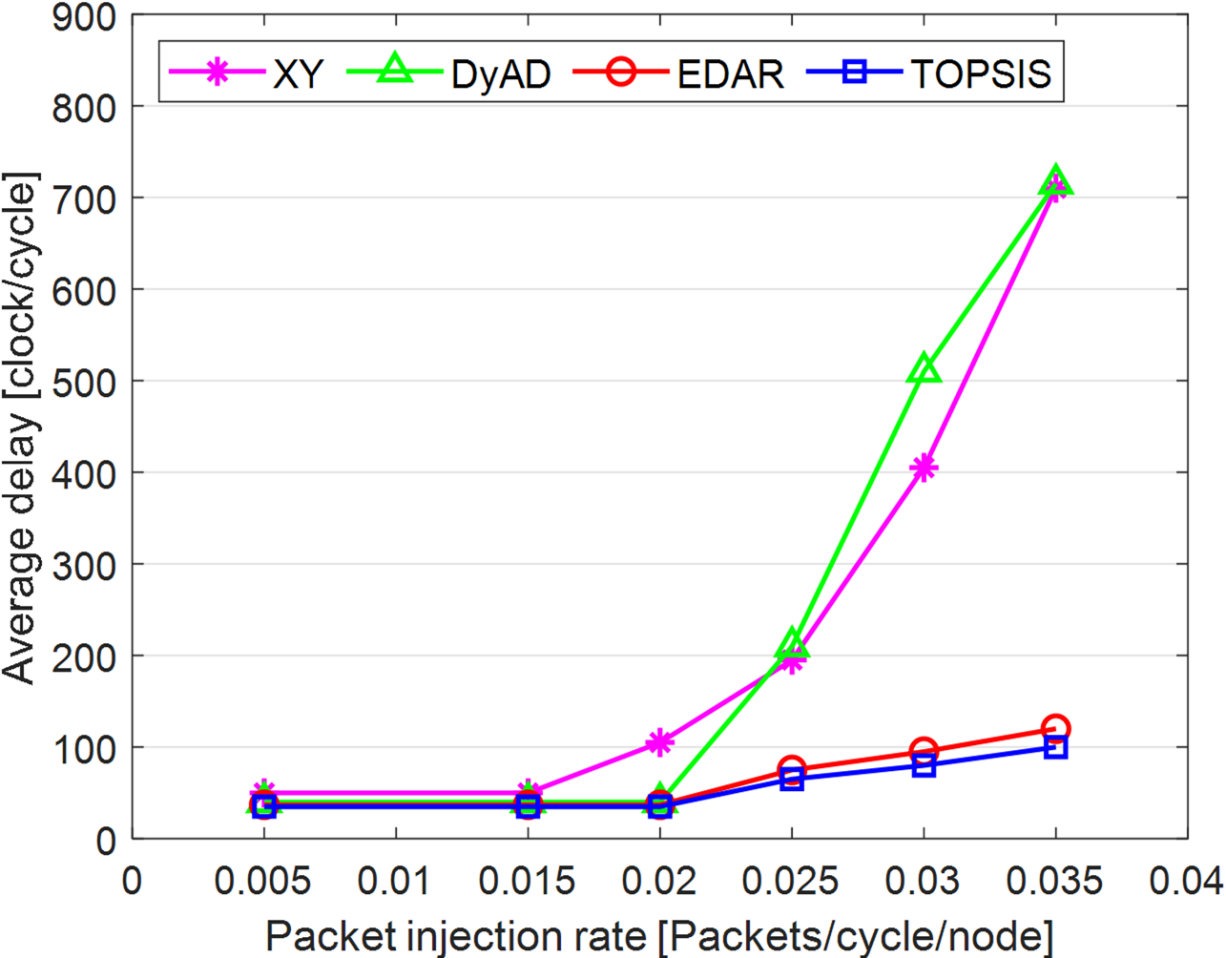
Analysis of the average delay for various algorithms under traffic load under failure conditions ranging from 5% to 20%.

**Fig. 13:**
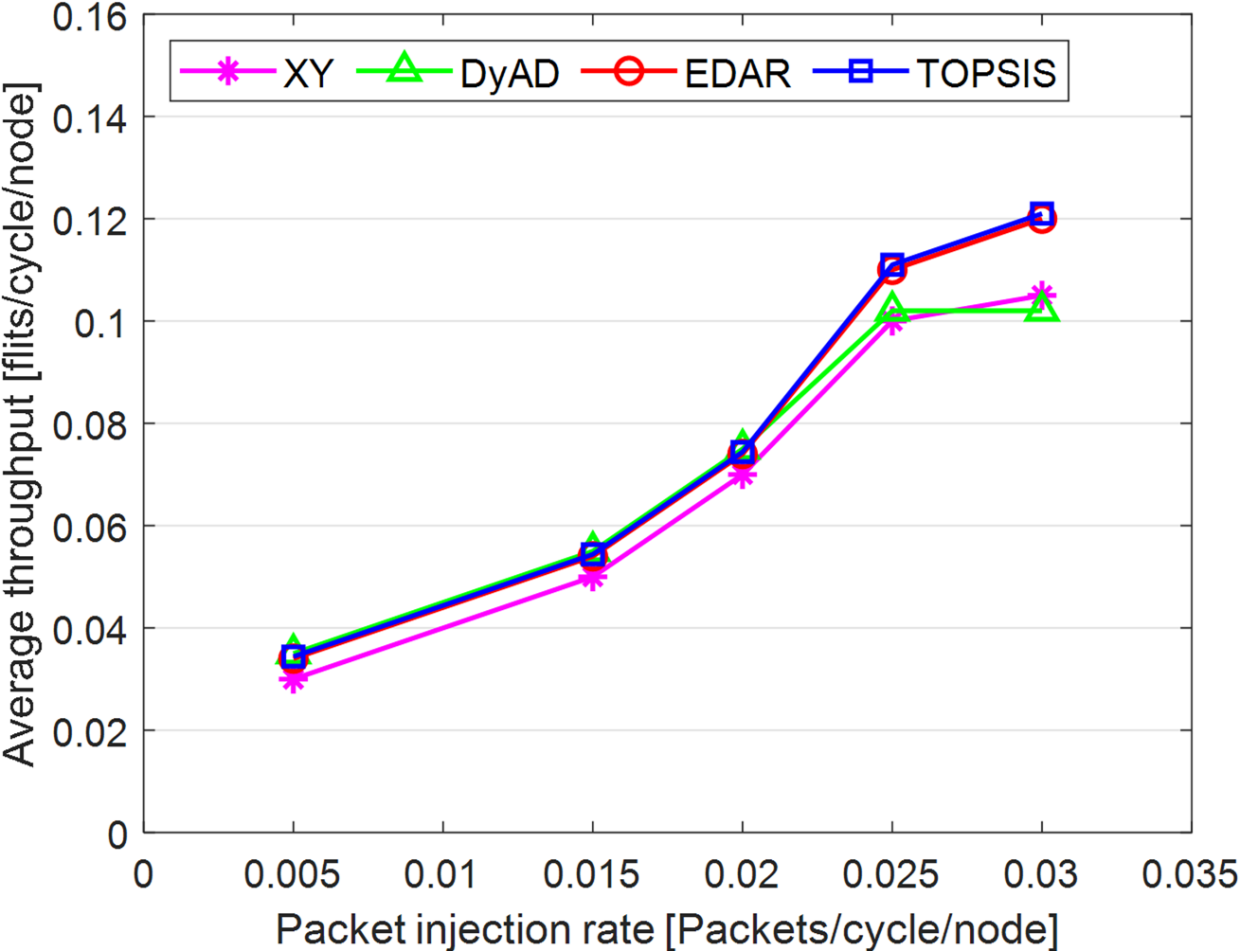
Analysis of the average throughput rate for several algorithms under traffic load with failure rates ranging from 5% to 20%.

**Fig. 14:**
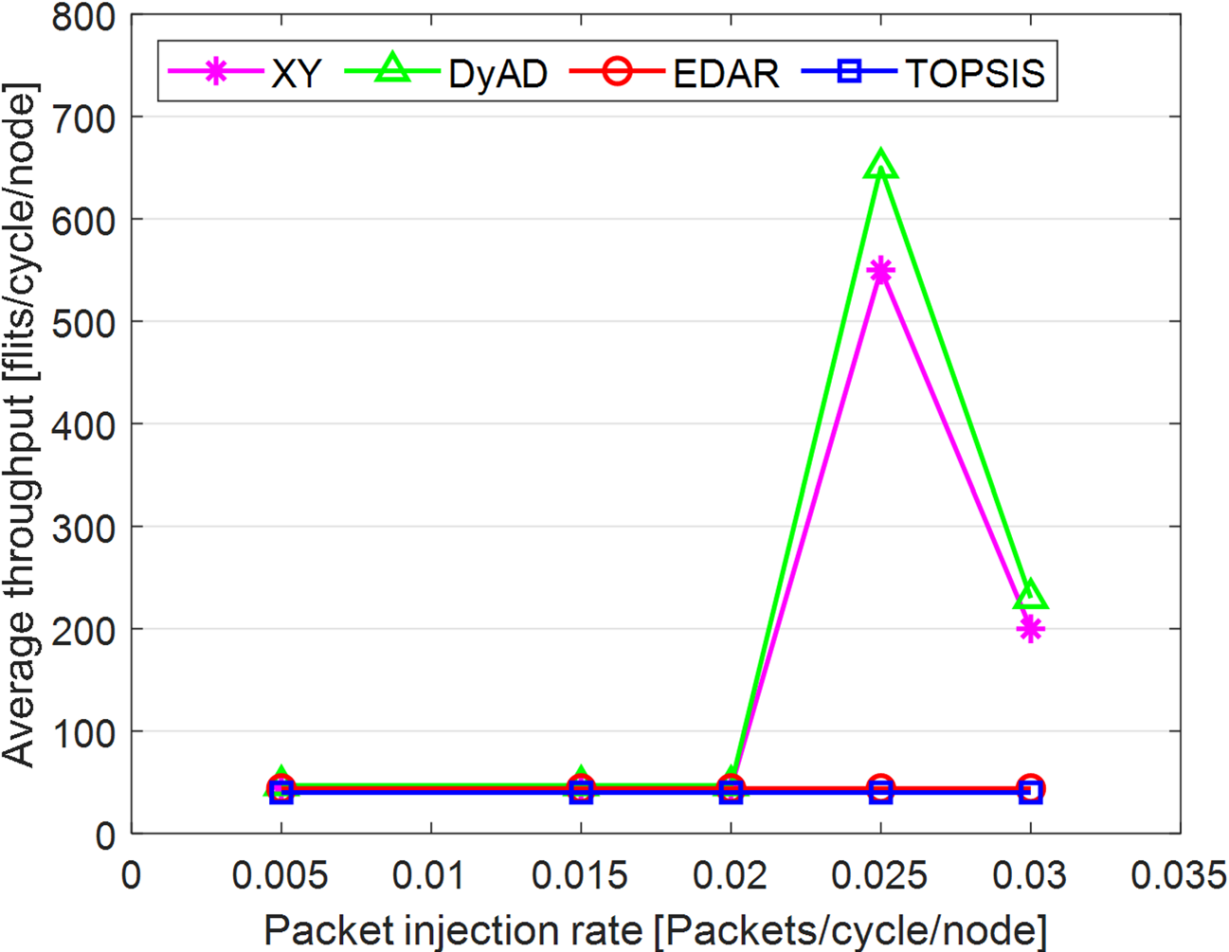
Analysis of the average delay for various algorithms under traffic load under failure situations ranging from 5% to 20%.

In Fig. 8, DyAD serves as the adaptive algorithm in these comparisons due to its capability to logically alternate between deterministic and adaptive routing contingent upon network congestion situations. This advantage enables the avoidance of crowded lines by exploring alternative routing algorithms, resulting in increased network throughput. Integrating deterministic and adaptive routing modes within a single network can guarantee the absence of deadlock and wandering. The XY method serves as the foundational algorithm to demonstrate the extent of operational power savings and fault tolerance in routing algorithms. The comparison outcomes are probabilistic owing to varying architectures. Table 11 presents the average throughput reduction outcomes under both fault-free and fault-tolerant scenarios. Figures 9, 10, 11, 12, 13 and 14 illustrate the outcomes of average throughput and delay under Random, Shuffle, and Transpose traffic loads in scenarios of 5% to 20% failure.

**Table 11:** Shows an average 5%–20% decrease in throughput rate under both non-faulty and defective conditions.

Algorithm	Failure (%)	Transpose		Shuffle		Random		Average delay (cycle)	Average throughput rate (flits/cycle)
		Average throughput rate (flits/cycle)	Average delay (cycle)	Average throughput rate (flits/cycle)	Average delay (cycle)	Average throughput rate (flits/cycle)	Average delay (cycle)		
TOPSIS	0	5.45	113.28	5.44	24.65	5.58	24.53	54.15	5.33
	5	5.35	110.96	5.34	24.15	5.47	24.06	53.06	5.23
	10	5.12	110.95	5.11	23.07	5.24	22.98	50.67	5.01
	15	4.42	90.57	4.41	19.75	4.52	19.68	43.33	4.32
	20	3.71	75.2	3.71	16.44	3.8	16.38	36.01	3.63
Average energy: 8.4278e-06j.s									
EDAR	0	5.16	383.32	5.39	94.4	5.15	227.32	235.01	4.93
	5	5.09	379.15	5.33	93.57	5.08	224.85	232.52	4.87
	10	4.91	364.49	5.14	89.96	4.89	216.16	217.54	4.69
	15	4.2	309.02	4.4	76.3	4.19	183.28	189.53	5.02
	20	3.46	250.76	3.63	61.96	3.45	148.75	153.82	3.32
Average energy: 8.5117e-06j.s									
XY	0	5.43	96.86	5.36	24.36	5.57	25.26	49.13	5.36
	5	3.79	65.94	3.74	16.66	3.88	17.27	33.29	3.74
	10	2	32.46	1.99	8.32	2.05	8.62	16.47	1.96
	15	1.02	14	1.02	3.72	1.04	4.05	7.26	0.99
	20	0.55	5.12	0.55	1.51	0.56	1.74	2.79	0.54
Average energy: 8.4734e-06j.s									
DyAD	0	5.46	33.15	5.45	19.1	5.49	25.79	20.47	5.45
	5	4.13	24.67	4.12	14.25	4.15	19.21	19.38	4.12
	10	2.53	14.49	2.52	8.42	2.54	11.31	11.41	2.52
	15	1.33	6.92	1.33	4.09	1.34	5.44	5.48	1.33
	20	0.75	3.19	0.75	1.96	0.75	2.55	2.56	0.75
Average energy: 8.5263e-06j.s									

In Fig. 9, under identical traffic load, the throughput of the DyAD algorithm ceases at a 30% injection rate due to the method’s adaptive architecture, subsequently continuing at a uniform rate. The XY algorithm exhibits a significant decline in throughput at a 40% injection rate. Under these settings, EDAR and TOPSIS exhibit identical slopes. In Fig. 10, with a packet injection rate exceeding 20%, the average delay under Random traffic load exhibits a considerable rise across all algorithms.

Under a 20% packet injection rate with Transpose traffic load, the throughput rate is enhanced for the three patterns: DyAD, TOPSIS, and EDAR, attributable to their adaptable architecture. This is not applicable to XY. The criteria depicted in Fig. 12 similarly apply to delays. At minimal packet injection rates, specifically between 5% and 15%, XY and DyAD exhibit the greatest delays, whilst EDAR and TOPSIS demonstrate the least delays. Under a packet injection rate below 30% with Shuffle traffic load, the three patterns DyAD, TOPSIS, and EDAR exhibit an increase in throughput attributable to their adaptable structure, as illustrated in Fig. 13. However, XY first undergoes a decline in throughput before stabilizing.

Typically, as the failure rate escalates, the DyAD and XY algorithms are unable to select pathways devoid of failures for flight transmission. Consequently, a substantial quantity of flights is forfeited, resulting in a major reduction in the number of incoming flights. Nonetheless, for TOPSIS, the average delay also grows, while the quantity of received flights remains constant.

As illustrated in Fig. 14, under Shuffle traffic conditions with a packet injection rate of up to 30%, all four algorithms exhibit comparably low delay rates, akin to the Transpose pattern. However, with XY and DyAD, this situation abruptly alters, and the patterns experience significant delays because to their intolerant structure, which progressively adjusts to the surroundings. The TOPSIS and EDAR algorithms exhibit identical and little delay without any alterations.

Under failure rates of up to 20%, the XY, DyAD, and EDAR models exhibit average throughput reductions of 43.49%, 51.98%, and 9.57%, respectively, while the TOPSIS model maintains a low failure rate of 9.30%. Nonetheless, as the failure rate escalates, the average time correspondingly rises. Nevertheless, the XY and DyAD algorithms exhibit a reduced delay rate owing to the exclusion of lost fleet counts.

The suggested method employs a multi-criteria decision-making process that incorporates QoS elements such as channel status, path priority weight, and neighbor buffer size, successfully identifying the most optimal way and greatly minimizing time. The suggested method selects channels with the highest priority and minimal congestion in the buffer, resulting in significant optimization of delivery rate and duration, as well as enhanced efficiency in the average delay rate component.

### Energy results

Tables 12, 13 and 14 illustrate that, across all traffic patterns and mesh sizes, the proposed TOPSIS method consistently exhibits a beneficial balance between power consumption and energy efficiency. In smaller 4 × 4 meshes, the disparity between methods is minimal; however, as the network expands to 8 × 8, the divergence increases, with TOPSIS exhibiting significantly lower $\:{E}_{flit}$ and $\:{E}_{packet}$. This signifies that the algorithm efficiently alleviates the energy costs usually linked to fault-induced delays and congestion. Moreover, although EDAR and DyAD sustain competitive throughput, their adaptive mechanisms result in increased switching and allocator activity, which is evidenced by elevated dynamic energy consumption. In contrast, TOPSIS effectively balances adaptability and stability, resulting in decreased total energy consumption without suffering leakage overhead. Key Observations:

**Table 12:** Orion configuration parameters applicable to all schemes.

Parameter	Value
Technology node	45 nm
Supply voltage (Vdd)	1.0 V
Clock frequency	100 MHz
Flit width	36 bits
Router ports	5
VCs / port	2
FIFO depth	8 flits
Mesh sizes	4 × 4, 8 × 8
Link length	Tile pitch

**Table 13:** 4 × 4 mesh average power and energy results with Orion 3.0.

Failure rate	Algorithm	P_avg (mW)	E_flit (nJ)	E_packet (nJ)
0%	XY	92.4	0.96	12.5
	DyAD	98.7	1.12	13.9
	EDAR	104.3	1.25	14.7
	TOPSIS	95.1	1.00	12.8
10%	XY	78.5	1.83	22.4
	DyAD	85.2	1.65	21.3
	EDAR	91.6	1.47	19.5
	TOPSIS	82.8	1.25	16.8
20%	XY	65.2	2.75	33.1
	DyAD	70.8	2.41	30.6
	EDAR	76.3	2.10	27.9
	TOPSIS	68.9	1.78	23.5

**Table 14:** 8 × 8 mesh average power and energy results with Orion 3.0.

Failure rate	Algorithm	P_avg (mW)	E_flit (nJ)	E_packet (nJ)
0%	XY	188.7	1.15	14.8
	DyAD	197.2	1.28	15.9
	EDAR	205.6	1.34	16.7
	TOPSIS	192.4	1.18	15.2
10%	XY	156.3	2.02	26.1
	DyAD	167.1	1.89	24.6
	EDAR	173.4	1.74	22.8
	TOPSIS	161.8	1.46	20.4
20%	XY	124.8	3.18	41.3
	DyAD	132.6	2.89	38.6
	EDAR	140.7	2.57	35.2
	TOPSIS	128.9	2.05	28.7

- Fault-free: TOPSIS achieves energy values close to XY, while EDAR incurs higher power due to constant adaptivity.
- With faults (≥ 10%): TOPSIS reduces $\:{E}_{flit}$ by ~ 15–20% compared to EDAR and ~ 25% compared to DyAD.
- Scalability: In 8 × 8 meshes, link energy dominates; TOPSIS lowers total energy by reducing unnecessary detours and retransmissions.

**Fig. 15:**
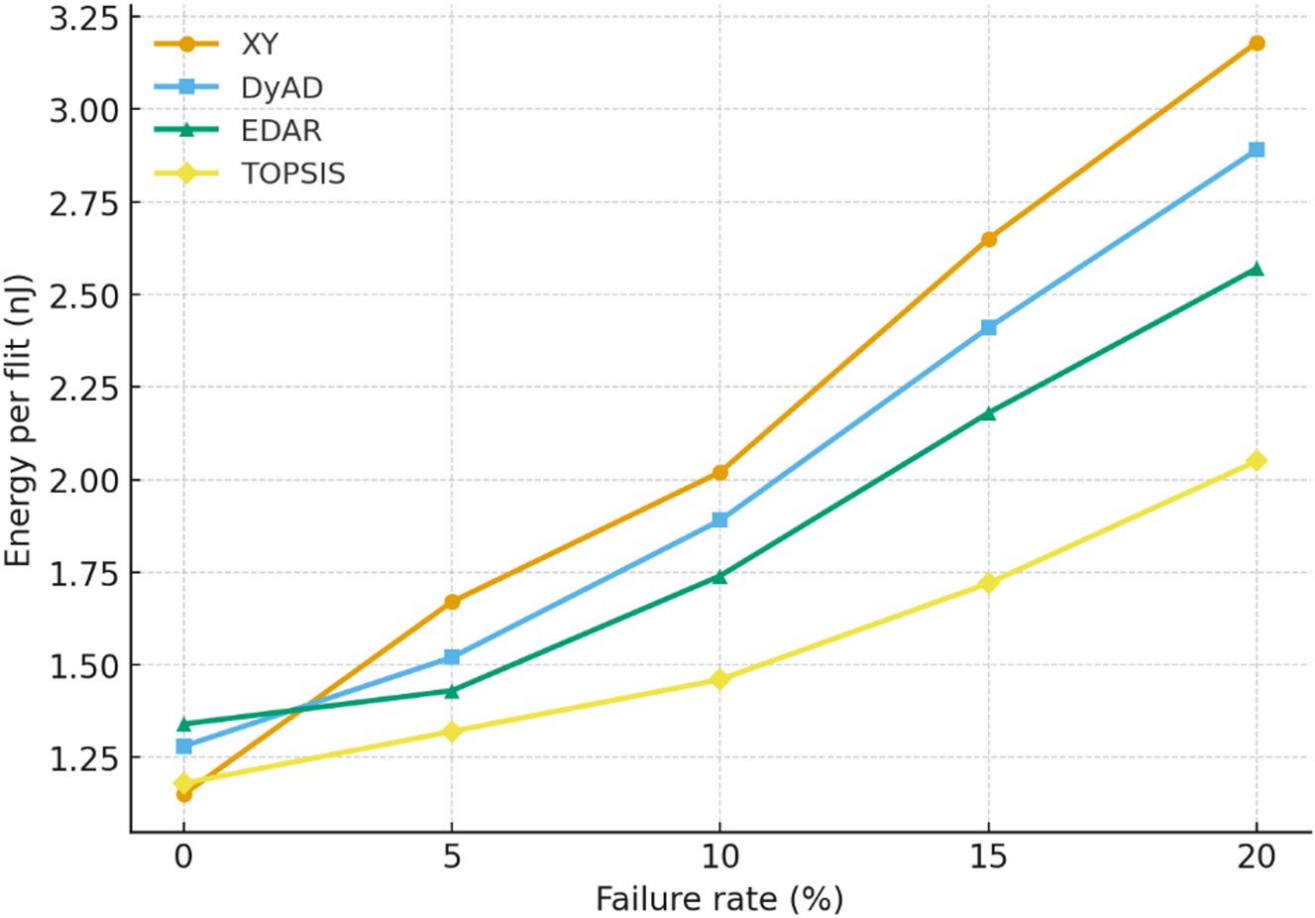
Energy per flit (nJ) versus failure rate (%) for the 8 × 8 mesh under random traffic conditions.

Figure 15 further demonstrates the resilience of the proposed technique by graphing energy per flit in relation to failure rates in an 8 × 8 mesh under random traffic conditions. As the failure rate escalates, all algorithms demonstrate increased energy expenditures resulting from extended pathways and retransmissions. The slope of the TOPSIS curve is markedly flatter, indicating superior energy efficiency under stress. XY routing demonstrates efficiency at 0% failure; however, its performance deteriorates sharply after 10% failures, indicating significant packet loss and retransmission costs. EDAR and DyAD exhibit elevated growth rates attributable to their adaptive yet less selective path exploration. Conversely, TOPSIS exhibits the minimal increase, diminishing inefficiencies in allocators and crossbars, and ensuring that energy consumption escalates more smoothly with fault severity.

### Hardware-level performance and overhead analysis

To express the area overhead of the algorithm, the EMBRACE NOC router^32^ is investigated. It has an online problem detection system and includes a monitoring module for each channel.

With deep learning-based sensor error compensation, it inspires data-driven approaches for error and noise estimation/correction^35^. Adaptive multipath and low-power routing for low-link IoT networks has a direct relationship with energy and multi-criteria weighting results^36^. The suggested algorithm resembles the structure and operation of solution^17^, differing solely in its decision-making approach. The solution^17^ has an area of 241 mm² and a power consumption of 291.2 mW. The propagation path of the error signal is not significantly critical at the system’s low clock frequency of 100 MHz, as it does not necessitate a clock cycle for propagation.

**Fig. 16:**
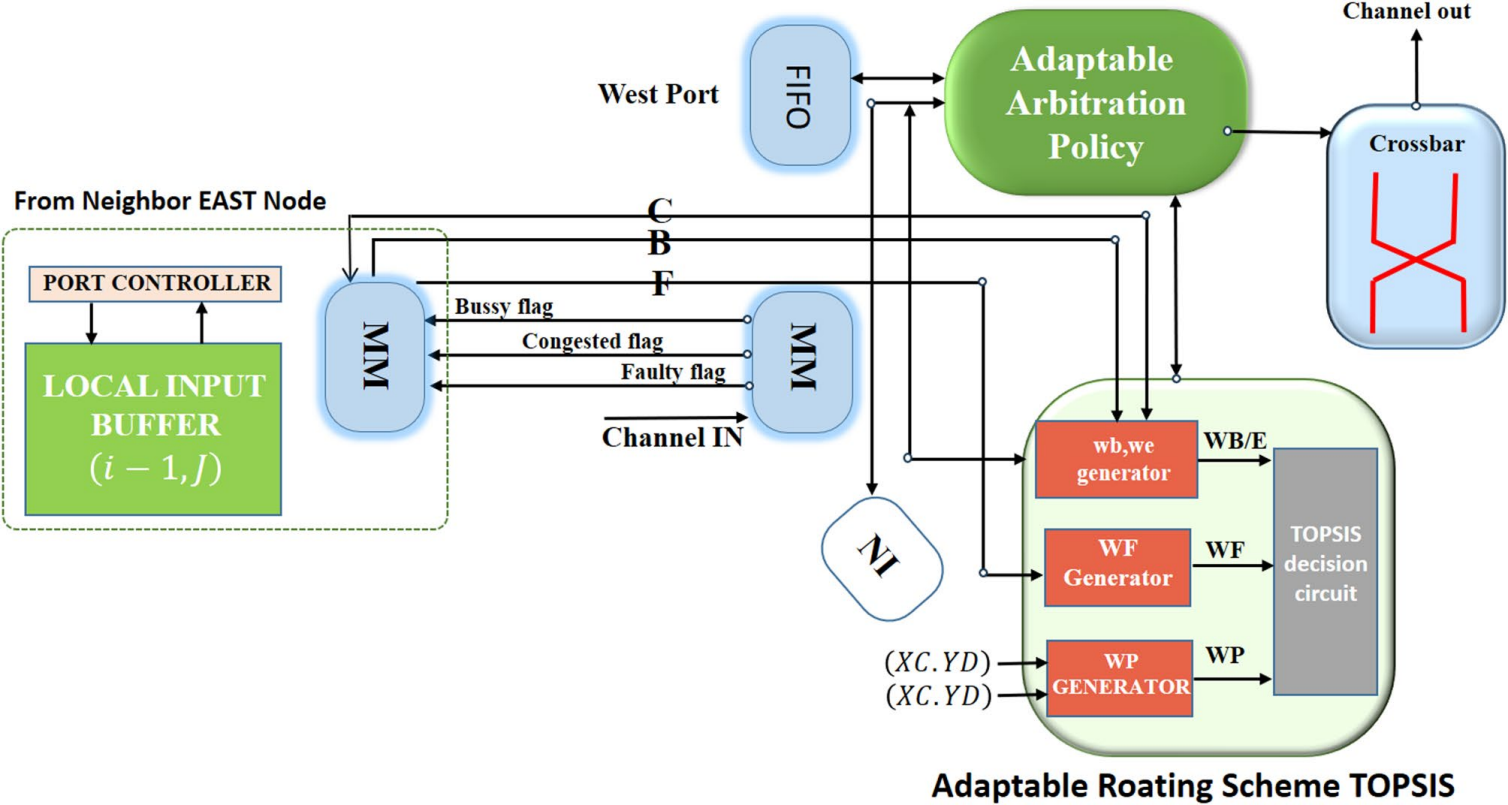
Structure of the TOPSIS adaptive router.

In instances of transient failures in the adjacent router, the packet is directed to an error-free route. If an error arises in a remote router upon the packet’s arrival, the problem is rectified, thus preventing the transmission of the error message from delaying the critical path and the operational frequency. Allocating 36 bits to the channel width is essential for receiving traffic and fault control signals, resulting in an acceptable cost of 8.5%. Consequently, the supplementary wire expense is often below 10%, indicating that it is not a hardware constraint; thus, the hardware-level overhead facilitates the preservation of system scalability. Figure 16 illustrates the configuration of the router. The comparator receives the weights of the path priority and channel status, the desired paths are designated, and ultimately, the TOPSIS decision algorithm is employed to determine the ranking and identify the optimal port as the most appropriate output path. The mean time complexity of the method depicted in Fig. 16 for computing the traffic weight, path weight, control signal reception time, and the average calculation time of the TOPSIS algorithm are 1.152E-06s, 1.541E-06s, 1.292E-05s, and 0.00217s, respectively, with an expected total of 10,000 cycles.

**Fig. 17:**
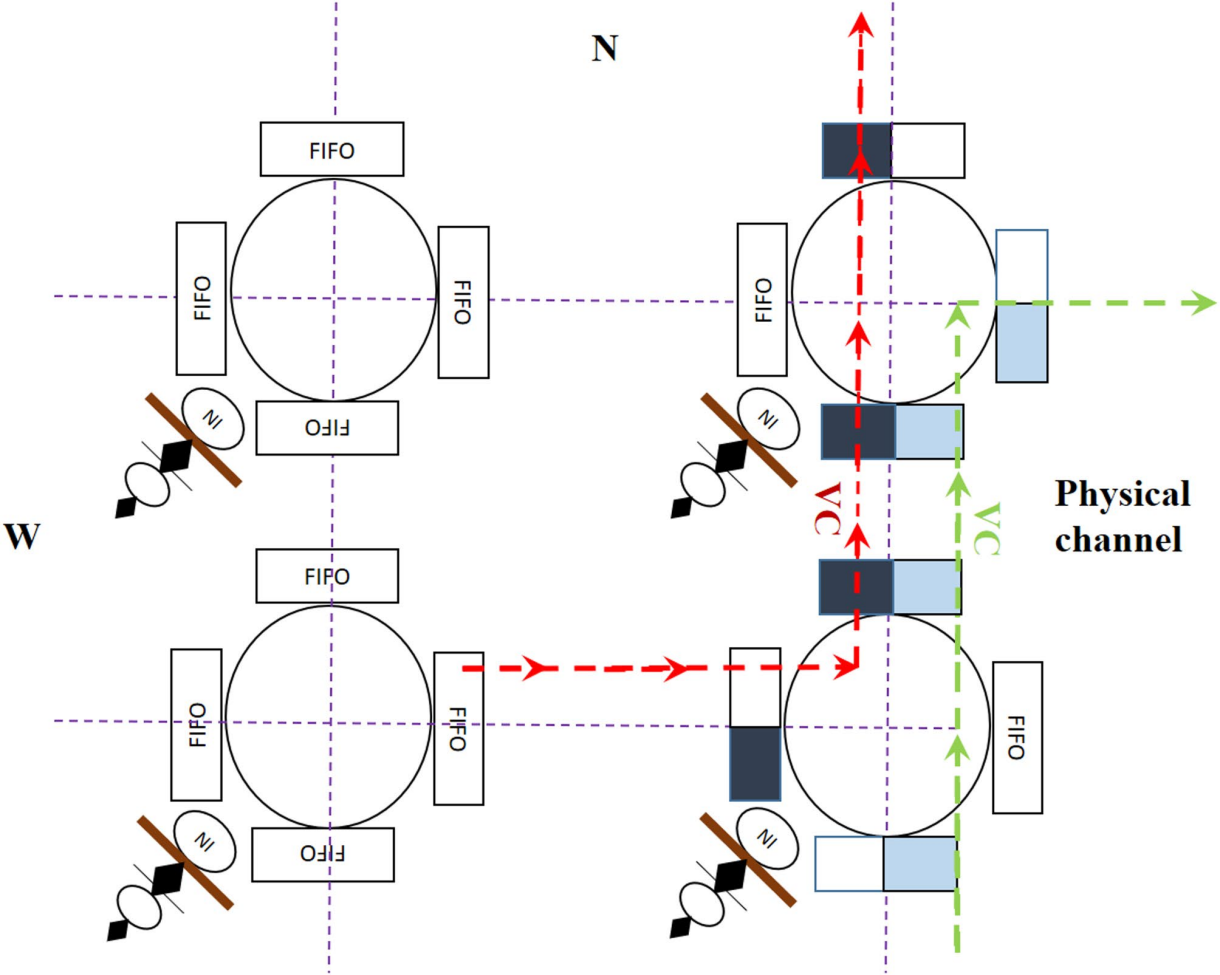
Virtual channels in the router.

This strategy involves the temporary storage of packets in VCs via a combination of first-in, first-out (FIFO) components. Figure 17 illustrates an instance of a round-robin policy for VCs. Each packet fills the channel upon reaching the common physical medium, which is partitioned into two VCs (green and red) according to the transport mechanism and facilitated by multiplexing. If another packet simultaneously demands the same channel, the physical channel is allocated between the packets. Consequently, the implementation of the adaptive arbitration policy, in conjunction with the VC, ensures that no transmitted packets are obstructed, thereby enhancing the system’s operating capacity. The distribution of buffers and available space from various VCs aids in routing decisions and diminishes the traffic load density at the node’s ingress port. Consequently, if insufficient space exists for the ingress port, node, or designated VC, the routing algorithm will select an alternative route to alleviate the traffic strain.

### Application-driven traffic

Representative application-based traffic mapped into 4 × 4 and 8 × 8 meshes was assessed as an addition to the synthetic models. As is customary, an H.264 decoding workload (media pipeline) and communication traces obtained from Blackscholes and Canneal (PARSEC) were employed. A location-aware mapping was used to position the tasks, the transmission rate was maintained using on/off propagation intervals, and packing was done according to the simulator’s defaults (wormhole flow control, variable width 36 bits). The warm-up and run measurement windows were identical to those in Sect. 4. Throughput and average delay (cycles) in the same packet injection locations utilized for the synthetic experiments are the metrics that are given.

The application-based outcomes on a 4 × 4 network with 0% error are shown in Table 15. In comparison to EDAR, TOPSIS lowers latency while matching or marginally improving XY/DyAD (1–2% difference) in Blackscholes, Canneal, and H.264. This effect is more noticeable for Canneal, where allocator/crossbar activity is increased by bursty and hotspot-prone traffic: TOPSIS reaches 5.35 times per cycle, 27.1 cycles, whereas EDAR reaches 5.19 times per cycle, 31.2 times per cycle, suggesting more stable path selection under conditions of transient congestion.

**Table 15:** 4 × 4 mesh, application-driven traffic, fault-free (0% links failed).

Benchmark	Algorithm	Throughput (flits/cycle)	Avg. delay (cycles)
Blackscholes	XY	5.42	23.1
	DyAD	5.40	21.7
	EDAR	5.18	24.9
	TOPSIS	5.41	22.0
Canneal	XY	5.12	33.8
	DyAD	5.27	28.4
	EDAR	5.19	31.2
	TOPSIS	5.35	27.1
H.264 decode	XY	5.36	26.7
	DyAD	5.44	24.1
	EDAR	5.22	27.9
	TOPSIS	5.45	23.5

The 8 × 8 network under 10% link fault is summarized in Table 16. XY is substantially degraded as network scale and variations make congestion worse, while DyAD marginally lessens it while still examining busy places. Although TOPSIS offers the maximum throughput and lowest latency for all workloads by simultaneously ranking path length, congestion stress, and error status, EDAR is still competitive. Compared to 5.01 times per cycle for EDAR, 2.68 times per cycle for DyAD, and 2.22 times per cycle for XY, TOPSIS, for instance, reaches 5.14 times per cycle, 39 cycles in H.264.

**Table 16:** 8 × 8 mesh, application-driven traffic, 10% link faults.

Benchmark	Algorithm	Throughput (flits/cycle)	Avg. delay (cycles)
Blackscholes	XY	2.15	85
	DyAD	2.62	68
	EDAR	4.95	46
	TOPSIS	5.08	41
Canneal	XY	1.98	102
	DyAD	2.47	79
	EDAR	4.72	55
	TOPSIS	4.96	49
H.264 decode	XY	2.22	78
	DyAD	2.68	64
	EDAR	5.01	44
	TOPSIS	5.14	39

### Transient/environmental evaluation

In the same injection region as in Sect. 4, three non-static sample situations are simulated on an 8 × 8 grid with random traffic:

- *Transient bursts*: $\:p=5\hspace{0.17em}\times\:{10}^{-4}$, $\:r=5\hspace{0.17em}\times\:{10}^{-3}$ (avg. bad burst ≈ 200 cycles, inter-burst ≈ 2000 cycles);
- *Thermal gradient*: steady $\:\varDelta\:T\approx\:\hspace{0.17em}30\circ\:C$ from a hot corner;
- *Voltage droop*: 5% of cycles flagged as droop windows system-wide.

Table 10 demonstrates that TOPSIS retains the maximum throughput and the lowest delay in all three transient scenarios; in particular, it improves throughput by approximately 3–4% and reduces delay by approximately 8–10% compared to EDAR. The difference with DyAD is more pronounced (throughput improvement of more than 30% and delay reduction of more than 20%), and XY exhibits the biggest decline when instabilities are present. Without altering the technology or architecture configuration, the superiority shown in Table 10 results from the decision matrix’s downgrade of unstable, hot, or dropped links and its quick return to healthy paths; as a result, the routing mechanism, not the modeling, is to blame for the discrepancies.

### Sensitivity to detection latency

Detector delay’s effect. The detection delay was increased from 1 to 8 cycles in order to test robustness to slower detectors. Despite the indications of error delay, health and hysteresis maintain decisions steady over time, as demonstrated by the results in Table 17, which reveal only a slight influence on TOPSIS: a decrease in throughput of ≈ 4–5% (5.08→4.85 changes per cycle) and an increase in delay of ≈ 3 cycles (41→44).

**Table 17:** Sensitivity to detection latency (8 × 8, 10% faults; throughput in flits/cycle, delay in cycles).

Detection latency (cycles)	Throughput (TOPSIS)	Delay (TOPSIS)
1	5.08	41
4	4.99	42
8	4.85	44

### Scalability to larger networks

The assessment process is repeated on a 16 × 16 network with random traffic in the same injection area to assess scalability. Under error-free and 10% link error regimes, the throughput (number of transmissions/cycle) and average delay (cycles) are given. The router’s microarchitecture and routing settings don’t change.

As the diameter and deflection pressure rise, Table 18 shows a rapid decline in XY; DyAD aids but still looks at dense areas. In the 10% error case, TOPSIS provides the maximum throughput and the lowest latency, but EDAR maintains its competitiveness with its congestion/error-aware ranking of alternatives. TOPSIS fits the 4 × 4 and 8 × 8 trends and stays near XY/DyAD in the error-free regime with very little overhead.

**Table 18:** 16 × 16 mesh, throughput/delay (random traffic).

Faults	Algorithm	Throughput	Avg. delay
0%	XY	4.72	31
	DyAD	4.81	28
	EDAR	4.63	33
	TOPSIS	4.78	29
10%	XY	1.42	128
	DyAD	1.88	103
	EDAR	3.96	71
	TOPSIS	4.21	64

Link energy dominates on bigger meshes is followed by energy scaling. TOPSIS maintains a lower $\:{E}_{\text{f}\text{l}\text{i}\text{t}}$ than EDAR/DyAD by eliminating hot links and shortening effective pathways.

### Weighting strategy and sensitivity analysis

Justification and Standardization. Path length, congestion/stress, and fault/health are the three criteria used in TOPSIS decision making. The weights ($\:{w}_{\text{p}\text{a}\text{t}\text{h}}$, $\:{w}_{\text{c}\text{o}\text{n}\text{g}}$, $\:{w}_{\text{f}\text{a}\text{u}\text{l}\text{t}}$) are normalized to 1.

The profiles are evaluated. (a) Equal (baseline), (b) Path Bias, (c) Congestion Bias, (d) Fault Bias, (e) Balanced-Resistant, and (f) Adaptive (Context-Aware), where the adaptive rule increases wfault, are the ones we look at. $\:{w}_{\text{f}\text{a}\text{u}\text{l}\text{t}}$ when $\:{w}_{\text{c}\text{o}\text{n}\text{g}}$ and health $\:h<0.9$ $\:{w}_{\text{c}\text{o}\text{n}\text{g}}$; The weights are renormalized at each period when stress > 0.5. The pipeline and decision kernel remain unchanged.

Tuning and Benchmark. At the same operational point, we report the relative change in delivered throughput and average latency against the Equal index. At 16 × 16 and at different loads, the trends remain consistent.

The performance is resilient to reasonable weight variations, as shown in Table 19. The adaptive law, which responds to temporary health or stress without overfitting a condition, produces the most stable gains, while the emphasis on defect/health aids in imperfect regimes (best fixed index).

**Table 19:** Sensitivity to weight profiles (8 × 8, 10% link faults). Changes vs. Equal profile.

Profile	($\:{w}_{\text{p}\text{a}\text{t}\text{h}}$, $\:{w}_{\text{c}\text{o}\text{n}\text{g}}$, $\:{w}_{\text{f}\text{a}\text{u}\text{l}\text{t}}$)	Δ Throughput	Δ Delay
Equal (baseline)	(0.33, 0.33, 0.34)	0.0%	0.0%
Path-biased	(0.50, 0.25, 0.25)	−2.1%	+ 3.2%
Congestion-biased	(0.25, 0.50, 0.25)	+ 0.8%	−1.6%
Fault-biased	(0.25, 0.25, 0.50)	+ 2.4%	−3.1%
Balanced-robust	(0.40, 0.30, 0.30)	+ 0.5%	−0.9%
Adaptive (context-aware)	≈(0.30, 0.34, 0.36)*	+ 3.1%	−5.0%

*Effective average over the run; weights are time-varying but normalized.

### Stress value calibration and granularity

This study’s suggested routers employ a FIFO depth of eight fleets. There is sufficient room for VC/SA arbitration without experiencing an early congestion at a “medium” threshold that is nearly half the capacity. In order to minimize flip-flopping between bins and give a hysteresis margin below the midway, 47% is used. The beginning of queue saturation, where backpressure and crossbar interference dramatically increase, is indicated by a “severe” threshold near 7.8 ≈ 87.5%. Thus, using hysteresis once more, 87% is utilized as a reasonable cutoff near 7.8.

The transitions employ the following bands to prevent oscillations: medium → severe when occupancy is $\:>0.87$, and low → medium when occupancy is > 0.47. The stress signal that TOPSIS receives is stabilized by these boundaries.

While the base is a 3-level stress (low, medium, and high), TOPSIS can take either (i) a continuous stress $\:{\text{s}}_{t}=\text{E}\text{W}\text{M}\text{A}\left({\text{t}}_{\text{o}\text{c}\text{c}}\right)\in\:\left[\text{0,1}\right]$. $\:{\text{s}}_{t}{\text{A}}_{\text{c}\text{o}\text{n}\text{g}}$(normalized) is the crowding criteria in the continuous case; the weights stay normal.

Table 20 shows that performance is resistant to moderate threshold modifications; switching from 47/87 to 45/85 or 50/90 lowers latency by one cycle and throughput by 1.2%. Small but steady gains are achieved by smoothing down brief bursts without oscillation using a continuous EWMA or finer 5-level quantization.

**Table 20:** Sensitivity to stress-threshold design (8 × 8, 10% faults).

Design	Thresholds (Low/Mod/Sev)	Throughput	Delay
Baseline (3-level)	< 0.47 / 0.47–0.87 / >0.87	5.08	41
3-level (tighter mid/high)	< 0.45 / 0.45–0.85 / >0.85	5.05	41
3-level (more conservative)	< 0.50 / 0.50–0.90 / >0.90	5.02	42
5-level (finer bins)	< 0.25 / <0.50 / <0.75 / <0.90 / ≥0.90	5.10	41
Continuous (EWMA, α = 0.2)	n/a (continuous sts_t)	5.12	40

## Conclusion

This study presents an adaptive routing algorithm that, after categorizing various paths between network nodes and assessing the congestion condition of adjacent nodes, prioritizes the available paths between source and destination nodes utilizing the TOPSIS multi-criteria decision-making method. Upon failure, an alternative routing algorithm with comparable QoS attributes is chosen.

The TOPSIS model’s advantage lies in its utilization of raw data and QoS factors, including channel traffic status, error detection, and path length, to assess routes according to quality-of-service standards. Consequently, by ensuring the efficacy of the routing algorithm during failures, it is feasible to prevent deadlocks in the network while delivering fault tolerance. The evaluation results provide enhanced performance metrics, including delay and throughput, relative to comparable methods, and in alignment with the architecture outlined in the EDAR technology, it facilitates the preservation of system scalability.

In future work, we will analyze the routing and fault detection strategy utilizing the independent quantities approach to select optimal paths through the lens of optimal control theory, a component of the minimum principle. We will propose a technique that enables traversal through defective ports by leveraging buffers under fault conditions.

## Data Availability

The custom code that implements the TOPSIS-based fault-tolerant, QoS-aware routing is publicly available at Zenodo: 10.5281/zenodo.17063199. It includes the transient/environmental fault models Gilbert-Elliott bursts, thermal gradient, and voltage-droop as well as the per-port stress-value update with EWMA+hysteresis. Additionally, a Supplementary File (noc_topsis_reference_impl.zip) containing the same snapshot is provided. The package includes scripts to replicate the reported throughput/latency/energy trends, the decision kernel, and a lightweight mesh NoC simulator. The code has no access limitations, requires Python ≥3.9, and is released under the MIT license.
